# Kappa-opioid receptor activation promotes depression-like behaviors by suppressing Pax6-dependent adult hippocampal neurogenesis

**DOI:** 10.1038/s41398-026-04230-z

**Published:** 2026-07-03

**Authors:** Hui-Sheng Peng, Ruo-Song Chen, Ru-Feng Ye, Cai-Yu Zhai, Xing-Cong Jiang, Pei-Pei Li, Jia-Yu Ye, Man-Man Han, Qian-Qian Fu, Yi-Quan Shen, Jing-Gen Liu, Chi Xu

**Affiliations:** 1 Department of Neurobiology and Acupuncture Research, Key Laboratory of Acupuncture and Neurology of Zhejiang Province, The Third Affiliated Hospital of Zhejiang Chinese Medical University, Hangzhou, 310053 China; 2 School of Pharmaceutical Sciences, Zhejiang Chinese Medical University, Hangzhou, 311400 China

**Keywords:** Depression, Molecular neuroscience

## Abstract

Accumulating evidence indicates that adult hippocampal neurogenesis (AHN) undergoes heterogeneous alterations in depression, yet the underlying mechanisms remain incompletely understood. In this study, we established a corticosterone (CORT)-induced mouse model of depression and combined pharmacological, molecular biological, and genetic approaches to investigate the mechanisms through which CORT suppresses AHN and induces depressive-like behaviors. Our results demonstrated that chronic CORT treatment led to depressive-like phenotypes in mice, including decreased sucrose preference and behavioral despair, accompanied by impaired AHN, manifested by a reduction in immature neurons (DCX⁺BrdU⁺) but an increase in proliferating cells (Ki67⁺). Further mechanistic studies revealed that CORT upregulates dynorphin A in the dentate gyrus (DG), leading to overactivation of the κ-opioid receptor (KOR). This subsequently inhibits the expression of Pax6 and its downstream targets Neurog2 and NeuroD1, thereby obstructing neuronal differentiation. The KOR antagonist nor-BNI effectively reversed both the depressive-like behaviors and AHN abnormalities induced by CORT. Moreover, overexpression of Pax6 alleviated depressive behaviors and restored neurogenesis, whereas knockdown of Pax6 was sufficient to induce depressive phenotypes and impair AHN. Our study unveils a central role of the KOR/Pax6 signaling axis in AHN suppression and depression pathogenesis, providing a theoretical foundation for antidepressant strategies targeting KOR or Pax6.

## Introduction

Major depressive disorder (MDD) is a psychiatric condition characterized by abnormalities across affective, cognitive, behavioral, and somatic domains, severely compromising quality of life [1]. According to reports from the World Health Organization, the number of individuals with depression has reached 380 million worldwide, making it a leading cause of disability [2]. However, the pathogenesis of depression is highly complex and multifactorial, involving genetic predisposition, environmental factors, and stress hormones, with adult hippocampal neurogenesis being closely implicated [3–6]. Numerous studies have demonstrated that MDD patients exhibit hippocampal volume reduction, structural abnormalities, and diminished neurogenesis, changes that can be reversed following antidepressant treatment [7, 8]. Despite decades of research, adult hippocampal neurogenesis in major depressive disorder shows heterogeneous alterations, yet the precise underlying mechanisms remain incompletely elucidated [9].

Adult hippocampal neurogenesis (AHN) refers to the process in which neural stem cells located in the subgranular zone of the dentate gyrus in adult mammals undergo asymmetric division, differentiate into neurons and glial cells, and subsequently migrate and integrate into existing hippocampal circuits [10]. This process plays a vital role in regulating mood and cognitive functions [11]. A growing body of research has demonstrated that significant suppression of AHN can lead to depressive-like behaviors, whereas its restoration can effectively alleviate depression [12]. Numerous studies have indicated that AHN plays a crucial role in the pathogenesis of corticosterone (CORT)-induced depression [13, 14]. In CORT-based rodent models of depression, AHN is markedly reduced, and antidepressant treatments that enhance AHN have been shown to reverse depressive behaviors [15]. These findings highlight a close association between AHN and CORT-induced depressive mechanisms, though the precise functional relationship warrants further investigation.

According to recent neuropharmacological studies on AHN, this process is regulated by multiple factors including opioids and their receptors, inflammatory cytokines, and neural circuits, which continuously modulate the proliferation, differentiation, and apoptosis of neural stem cells in the dentate gyrus [16–18]. The κ-opioid receptor (KOR), a subtype of the opioid receptor family, is widely expressed throughout the central nervous system and binds to the endogenous neuropeptide dynorphin to regulate diverse neurophysiological activities [19]. Existing evidence suggests that the dynorphin/KOR system is involved in the pathophysiology of various psychiatric disorders, including depression, anxiety, and addiction [20–22]. Preclinical studies have shown that KOR agonists induce significant negative emotional states such as depression, anxiety, and aversion, whereas KOR antagonists produce sustained antidepressant effects [23, 24]. In vitro studies further demonstrate that KOR agonists markedly inhibit the differentiation of neural stem cells into neurons, an effect that can be reversed by KOR antagonists [25]. Moreover, compared to other opioid receptors, KOR is expressed at relatively higher density in the hippocampus [26]. Based on these findings, it has been hypothesized that AHN may interact with the dynorphin/KOR system in the pathogenesis of depression. However, the underlying mechanisms remain unclear.

Paired box protein 6 (Pax6) is a highly conserved transcription factor among vertebrates and is primarily expressed in the adult subgranular zone of the hippocampal dentate gyrus and the subventricular zone [27]. Pax6 is present in neural stem cells (NSCs) throughout all stages of neurogenesis: embryonic, postnatal, and adult. It participates in and regulates a core transcriptional network that controls the balance between neurogenesis and stem cell self-renewal in a tightly dose-dependent manner [28]. Downstream targets of Pax6 include two basic helix-loop-helix (bHLH) transcription factors, Neurogenin 2 (Neurog2) and Neurogenic differentiation 1 (NeuroD1), which are expressed during neuronal differentiation of NSCs. Neurog2 is mainly expressed in early progenitors and promotes NSC proliferation and differentiation by guiding the production and expansion of adult dentate gyrus cells [29, 30]. NeuroD1 is predominantly expressed from the late progenitor to immature neuron stages and is responsible for the differentiation and survival of granule neurons [31]. Our previous in vitro studies indicated that the expression levels of Pax6, Neurog2, and NeuroD1 in NSCs are negatively regulated by the dynorphin/KOR system [25]. However, whether aberrant AHN in depression is associated with dysregulation of Pax6, Neurog2, and NeuroD1, and the underlying mechanisms involved, require further investigation.

## Materials and methods

### Animals

C57BL/6J mice were obtained from the Shanghai Laboratory Animal Center, Chinese Academy of Sciences. Transgenic *Nes*-CreER^T2^ mice (full strain name: C57BL/6-Tg(Nestin-Cre/ERT2)KEisc/J; RRID: IMSR_JAX:016261) and *Oprk*^*LoxP/LoxP*^ mice (full strain name: B6;129-Oprk1tm2.1Kff/J; RRID: IMSR_JAX:030076) were acquired from The Jackson Laboratory (USA). All mice were six weeks old at the beginning of each experiment. Induction of Cre recombinase was performed following the original study via intraperitoneal injection of tamoxifen (Merck, Germany) at a dose of 180 mg/kg/day for four consecutive days. All animals were housed under standard conditions (12-hour light-dark cycle, 24 ± 2 °C) with ad libitum access to food and water at the Laboratory Animal Center of Zhejiang Chinese Medical University, which is accredited by the Association for Assessment and Accreditation of Laboratory Animal Care (AAALAC). All mice were euthanized immediately upon completion of the experiments. All experimental procedures were approved by the Animal Ethics Committee of Zhejiang Chinese Medical University (Approval No.: IACUC-20220328-09) and were conducted in accordance with the National Institutes of Health Guide for the Care and Use of Laboratory Animals (NIH Publication No. 80-23).

### Anesthesia and analgesia

Mice were anesthetized with isoflurane (3% for induction, 1.5% for maintenance in oxygen) for routine procedures. For stereotaxic surgical procedures requiring prolonged precision manipulation typically exceeding 30 min, sodium pentobarbital (70 mg/kg, i.p.) was used to provide stable surgical-plane anesthesia for the full duration of the procedure. A non-opioid analgesic protocol was employed for all survival surgical procedures. Opioid analgesics were specifically excluded from the perioperative regimen to avoid pharmacological interference with the κ-opioid receptor signaling pathway under investigation. Meloxicam (4 mg/kg, s.c.) was administered 30 min prior to surgery as a pre-emptive analgesic, and the incision site was infiltrated with 0.3% bupivacaine prior to the first incision to provide local analgesia. Post-operatively, meloxicam (4 mg/kg, s.c.) was administered once daily for 3 consecutive days. Animals were maintained on a thermostatically controlled heating pad during surgery and throughout the recovery period and were monitored daily for signs of pain or distress until full recovery.

### Corticosterone-induced model of depression

The depression model was established based on a previously reported protocol with minor modifications [14]. Briefly, corticosterone (CORT, purity >98%, 235135, Merck) was dissolved in sterile distilled water containing 0.1% DMSO and 0.45% β-cyclodextrin to achieve a final concentration of 70 μg/mL. The solution was stored in light-protected bottles and provided ad libitum, with fresh solution replenished every three days. Mice in the experimental group received the CORT-containing solution for 21 consecutive days, while the control group received the vehicle solution (0.1% DMSO and 0.45% β-cyclodextrin in sterile water) only.

### Plasma corticosterone levels

To evaluate the response of the HPA axis to acute stress in the chronic CORT model, mice were subjected to a 1-minute swim stress during which they were placed in a beaker filled with room-temperature water. Orbital blood samples were collected 5 min post-stress into EDTA-coated tubes to prevent coagulation. The blood was then centrifuged at 2000 rpm for 5 min to isolate plasma, which was stored at −20 °C until analysis. Corticosterone levels were assessed using a commercial ELISA kit (K014-H1, Arbor Assays).

### Sucrose preference test (SPT)

The SPT was performed as previously described with minor modifications. Briefly, after acclimation to individual housing, mice were first habituated to two identical drinking bottles for 3 days, followed by 12 h of water deprivation. During the test, two bottles—one containing 2% (w/v) sucrose solution and the other plain water—were simultaneously provided for 2 h of free access. Sucrose preference was calculated using the following formula: Sucrose preference (%) = [Sucrose consumption / (Sucrose consumption + Water consumption)] × 100.

### Tail suspension test (TST)

The TST was performed as follows: approximately 2 cm from the tip of the mouse tail was secured with adhesive tape, and the animal was suspended head downward in the experimental apparatus (50 cm above the floor) for a total of 6 min. Immobility was defined as the absence of any initiated movement, including passive swinging. The total immobility time during the final 4 min was recorded, while behavior during the initial 2-minute acclimation period was excluded from analysis. The experimenter was blinded to the group allocation throughout the testing.

### Forced swimming test (FST)

The FST was conducted by placing mice in a transparent glass cylinder (20 cm in height, 14 cm in diameter) filled with water to a depth of 16 cm (water temperature maintained at 25 °C ± 1 °C). Each animal was allowed to swim freely for 6 min. The first 2 min were considered an acclimation period, and behavior during the final 4 min was recorded and analyzed. Immobility time, defined as passive floating with only minimal movements necessary to keep the head above water (such as slight paddling with a single paw), was interpreted as behavioral despair. The experimenter performing the test was blinded to the experimental group assignments.

### Microdissection of the mouse dentate gyrus

The DG was dissected from mouse brains according to a previously described protocol [32]. Briefly, following extraction of the whole brain using 20 mL of ice-cold Hank’s Balanced Salt Solution (HBSS, 14175095, Thermo Fisher Scientific, Waltham, MA, USA), coronal brain slices were trimmed using a double-edged razor blade and transferred to a 6-cm dish containing 5 mL of HBSS. Under a dissecting microscope, DG tissue fragments were carefully isolated and collected in 10 mL of HBSS, with all procedures performed on ice. Finally, dorsal (bregma −1.0 to −2.5 mm) and ventral (bregma −2.5 to −4.0 mm) DG tissues were obtained using an NX70 cryostat (Thermo Fisher Scientific).

### Quantitative reverse transcription PCR

Total RNA was extracted from DG tissue samples using Trizol reagent (Cat. No. 15596026, Thermo Fisher Scientific, Waltham, MA, USA). cDNA was synthesized from 1000 ng of total RNA using the PrimeScript™ RT-PCR Kit (Cat. No. RR014, Takara Bio, Shiga, Japan). Quantitative PCR (qPCR) was performed strictly in accordance with the manufacturer’s instructions using the FastStart Universal SYBR® Green Master PCR Kit (Cat. No. 4913850001, Roche, Basel, Switzerland). Each sample was run in triplicate. Actin was used as the reference gene for normalization of expression levels. Analysis of miRNA expression was performed using miRCURY LNA miRNA PCR Assays (Qiagen, Germany) with U6 as a reference gene. The cycle threshold (CT) values were acquired and analyzed using the Roche 384 Real-Time PCR System software, and relative mRNA expression was calculated using the 2^−ΔΔCT^ method. Primer sequences are listed in Supplementary Table S1.

### Western blot assay

All mice included in this study completed the entire behavioral testing paradigm. Following euthanasia, the dentate gyrus was dissected and homogenized, and the protein concentration of the supernatant was determined using a bicinchoninic acid (BCA) protein assay kit (Abcam, UK) in strict accordance with the manufacturer’s instructions. The protein concentrations of all samples were normalized, and a total of 10–20 μg protein per lane was loaded to mitigate the impact of membrane saturation on the accurate quantification of reference proteins ^28, 29^. Samples were mixed with loading buffer containing 2% SDS, 100 mM dithiothreitol, 10% glycerol, and 0.25% bromophenol blue, followed by separation via 10% SDS-PAGE and subsequent transfer onto a polyvinylidene fluoride (PVDF) membrane (Merck, Germany). Signals were developed using ECF chemiluminescence substrate (GE Healthcare, UK) and captured with an ImageQuant LAS 4000 imaging system (GE Healthcare). Band intensity was quantified using Quantity One 1D analysis software (Bio-Rad, USA). Antibody information is provided in Supplementary Table S2. The uncropped membrane images for all representative blots shown in the main and supplementary figures are provided as a supplementary material.

### Bromodeoxyuridine (BrdU) labeling, immunohistochemistry, and in vivo cell counting

To evaluate neurogenesis, mice received intraperitoneal injections of bromodeoxyuridine (BrdU; 150 mg/kg/day; Cat. 19–160, Merck, Darmstadt, Germany) for three consecutive days and were sacrificed at designated time points. Under deep anesthesia, transcardial perfusion was performed using ice-cold saline, followed by fixation with 4% fresh paraformaldehyde (PFA). The brains were post-fixed in 4% PFA for 4 h and then cryoprotected overnight in 30% sucrose solution at 4 °C. Using a cryostat (NX70, Thermo Fisher Scientific, Waltham, MA, USA), 30-μm-thick coronal sections were prepared encompassing the dorsal dentate gyrus (DG; bregma −1.0 to −2.5 mm) or ventral DG (bregma −2.5 to −4.0 mm). Every sixth section was selected for immunohistochemical staining. Sections were mounted on gelatin-coated slides and processed for immunofluorescence. After blocking with 10% normal goat serum in Tris-buffered saline containing 0.1% Tween 20 (TBST) for 1 h at room temperature, they were incubated with primary antibodies overnight at 4 °C. After washing steps performed in the dark, sections were incubated with fluorescence-conjugated secondary antibodies for 1 h at 37 °C in a water bath. Images were acquired using a Zeiss Axioscan 7 fully automated slide scanner (Carl Zeiss, Oberkochen, Germany). BrdU-positive cells were quantified on each section using the “Cell Counter” plugin in ImageJ software (NIH, Bethesda, MD, USA). The total number of fluorescently labeled cells was estimated by multiplying the average number of positive cells per section by the total number of 30-μm sections representing the dorsal or ventral DG. For each group, at least five sections were averaged for intergroup comparisons. Antibodies against BrdU were purchased from Abcam (Cambridge, UK) and Thermo Fisher Scientific (USA); detailed information is provided in Supplementary Table S2.

### Implantation of guide cannula and microinjection

Under anesthesia as described above, the scalp was incised to expose the skull surface. After removing the periosteum, bilateral holes were drilled, and a 26-gauge guide cannula was implanted into the DG of the hippocampus (coordinates: AP: −2.2 mm, ML ± 1.2 mm, DV −2.0 mm relative to bregma). The cannula was secured to the skull using stainless steel screws and dental cement. Animals with misplaced cannulas were excluded from analysis. Microinjections were performed using a 31-gauge internal injection cannula connected to a 10 µL Hamilton micro-syringe (KD Scientific microinjection pump system, USA). The internal injection cannula extended 1 mm beyond the tip of the guide cannula to prevent clogging. For κ-opioid receptor (KOR) activation or inactivation experiments, bilateral infusions of either 0.5 µL U50,488H (10 µg/µL in saline, administered 20 min prior to behavioral tests, with repeated dosing) or 0.5 µL nor-BNI (10 µg/µL in saline, administered as a single injection 24 h before behavioral testing) were delivered into the DG. All substances were infused at a rate of 0.2 µL/min, and the injection needle was left in place for an additional 2 min after each infusion to ensure adequate diffusion. Data were retained for analysis only when injection sites were confirmed to be accurate.

### Virus microinjection

Under anesthesia as described above, bilateral burr holes (0.5 mm in diameter) were drilled into the skull. A total volume of 500 nL of viral suspension (250 nL per side) was injected into the ventral dentate gyrus (stereotaxic coordinates: AP: −3.3 mm, ML: ±2.3 mm, DV: −2.1 mm relative to bregma) at a constant rate of 100 nL/min. The injection needle was left in place for 8 min following infusion to ensure adequate viral diffusion.

The following adeno-associated virus (AAV) constructs (BrainVTA, China) were used in this study:

rAAV2/9-GAG-DIO-DTA-mCherry-WPREs-hGH polyA

rAAV2/9-GAG-DIO-mCherry-WPREs-hGH polyA

rAAV2/9-EF1a-DIO-*Pax6*-EGFP-WPREs-hGH polyA

rAAV2/9-EF1a-DIO -EGFP-WPREs-hGH polyA

rAAV2/9-CMV-DIO-NLS-hSaCas9-NLS-2×HA-bGH-polyA-U6-SasgRNA1(*Pax6*)

rAAV2/9-CMV-DIO-mCherry-WPREs-hGH polyA

rAAV2/9-Nestin-Cre-WPREs

### Sample size estimation

For sample size estimation in behavioral experiments, assuming an effect size of Cohen’s d = 1.5, a significance level of α = 0.05, and a statistical power of 0.80, a minimum of n = 5 per group was required for two-group comparisons (two-tailed unpaired t-test). For 2 × 2 factorial designs analyzed by two-way ANOVA, sample sizes were increased to n = 8–15 per group to ensure sufficient power to detect interaction effects. For molecular and histological analyses (Western blot, immunohistochemistry, and qRT-PCR), n = 5–6 per group was used based on the typically larger effect sizes and lower variability of these measurements.

### Randomization

Prior to each experiment, mice were assigned to experimental groups using a random number table. Briefly, each mouse was assigned a sequential identification number upon arrival, and a random number table was used to allocate animals to specific treatment groups, ensuring unbiased group assignment. For experiments involving stereotaxic surgery (cannula implantation or viral injection), randomization was performed after surgery and a 7-day recovery period but before the onset of CORT or vehicle administration, so that any variability introduced by the surgical procedure was evenly distributed across groups. Body weights were recorded at the time of randomization to confirm that no significant baseline differences existed between groups.

### Blinding

Blinding was implemented at the outcome assessment stage. During data acquisition, all samples and animals were coded by a researcher who was not involved in data analysis; the codes were not revealed until all measurements were completed. Specifically, behavioral tests were video-recorded and scored by an investigator blinded to group allocation. For immunofluorescence analysis, coded brain sections were imaged and quantified by a blinded observer using standardized criteria. Western blot band intensities were quantified from coded membrane images using Quantity One 1D analysis software (Bio-Rad Laboratories, USA). Complete blinding during the experimental procedures (e.g., drug preparation and administration) was not feasible, as different treatment protocols required distinct handling.

### Statistical analysis

All data are presented as the mean ± standard error of the mean (SEM). The sample size (n), indicating the number of independent experimental subjects per condition, is specified in the corresponding figure legends. Statistical analyses were performed using GraphPad Prism software (version 10.0). Normality of data distribution was assessed using the Shapiro-Wilk test, and homogeneity of variance was evaluated using the F-test (for two-group comparisons) or Brown-Forsythe test (for multi-group comparisons). All datasets met the assumptions of normality and equal variance, justifying the use of parametric tests (unpaired t-test and ANOVA). In cases where the assumption of equal variance was not met, Welch’s correction was applied. Specific statistical methods are detailed in the figure legends. Comparisons between two groups were analyzed using two-tailed unpaired Student’s t-tests. For comparisons among multiple groups, one-way or two-way analysis of variance (ANOVA) was applied according to the experimental design. For 2 × 2 factorial designs, we performed two-way ANOVA followed by a priori planned pairwise comparisons using uncorrected Fisher’s LSD test. For Fig. S1C and S1D, which included a positive control group, one-way ANOVA followed by Tukey’s post hoc test was used. Statistical significance was defined as follows: *P < 0.05, **P < 0.01, and ***P < 0.001. Detailed statistical results for all comparisons, including test statistics (F or t values), degrees of freedom, and exact P values, are provided in Supplementary Table S3.

## Results

### Corticosterone induces depressive-like behaviors and suppresses AHN

**Fig. 1:**
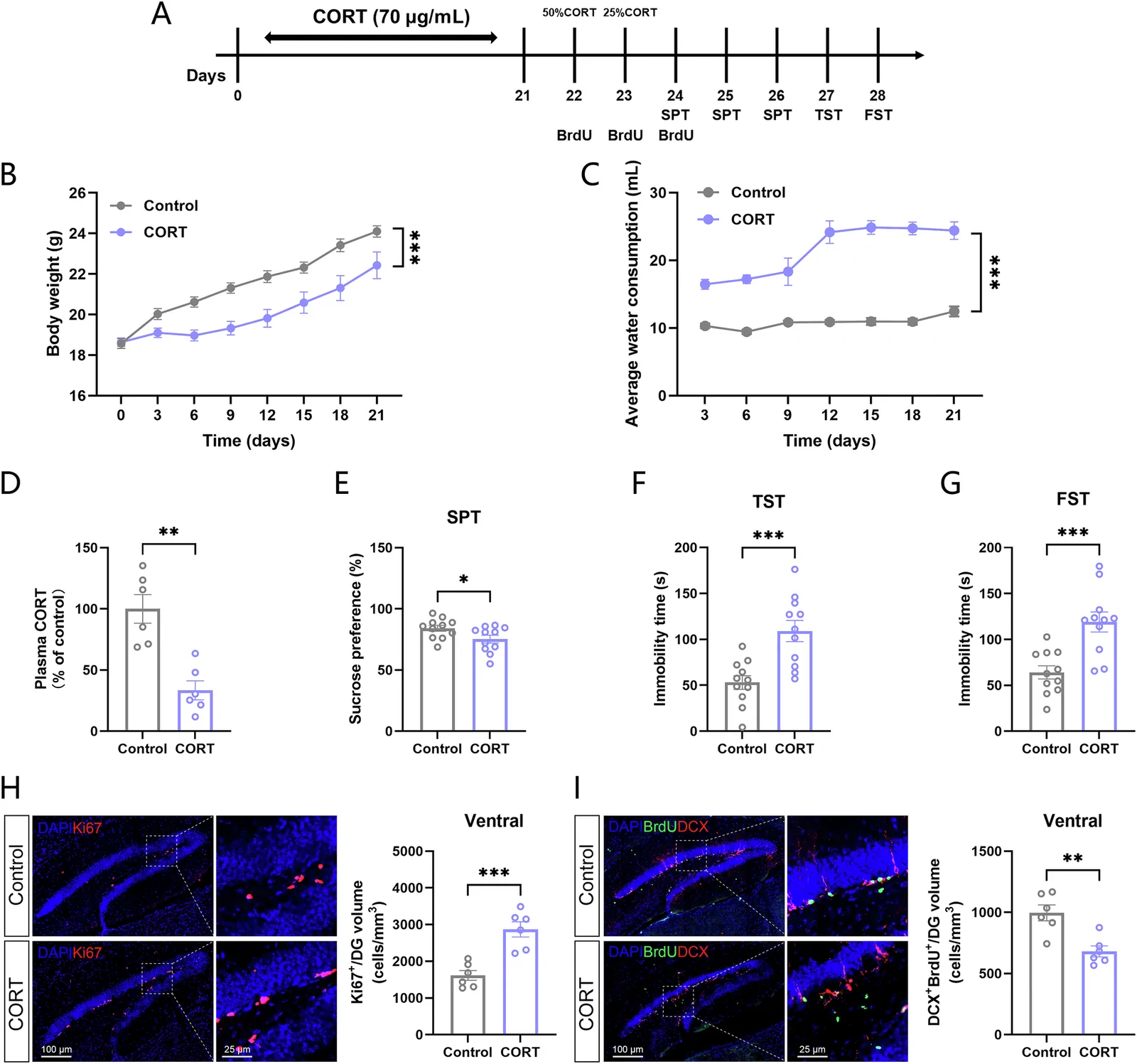
Depression-like behaviors, the differentiation and proliferation of neural stem cells in hippocampal dentate gyrus in mice with CORT treatment. **A** The experimental timeline of CORT treatment and three behavioral tests (SPT, TST, FST). **B**, **C** The body weight and average water consumption of mice were recorded every 3 days. n = 11. **D** Plasma corticosterone levels were quantified in both CORT-treated and control mice following FST. n = 6. **E**–**G** Sucrose preference (SPT, E) and immobility time (TST, F and FST, G) were assessed in CORT-treated and control mice on the indicated days of the experiment. n = 11. **H**, **I** Representative immunofluorescence images of Ki67^+^ (H) and BrdU^+^/DCX^+^ (I). The quantification of BrdU^+^/DCX^+^ immature neurons and Ki67+ proliferative neural stem cells in the ventral DG, calculated as the number of positive cells per mm^3^ volume of the DG in CORT-treated and control mice. n = 6. Scale bars: 100 μm for the entire DG and 25 μm for local enlarged panels. *P < 0.05; **P < 0.01; ***P < 0.001; error bars represent SEM. Statistical analysis was performed using two-way ANOVA in B and C, and unpaired t-test in D-I.

To investigate whether CORT induces depressive-like behaviors in mice, animals were provided with CORT solution ad libitum for 21 days, followed by a two-day gradual dose reduction to avoid withdrawal rebound effects (Fig. 1A). Depressive-like behaviors were assessed using SPT, TST, and FST. Body weight and fluid consumption were measured every three days during the treatment period. Reduced weight gain and polydipsia are characteristic of chronic CORT administration (Fig. 1B and C). The results showed that CORT-treated mice exhibited significantly lower weight gain and higher water intake compared to control mice. Another hallmark of prolonged CORT exposure is the atrophy of the hypothalamic-pituitary-adrenal (HPA) axis, which disrupts endogenous CORT secretion and leads to a pronounced decrease in plasma CORT levels after cessation of treatment [33] (Fig. 1D). CORT-treated mice displayed significant depressive-like phenotypes, including a reduced sucrose preference score in the SPT and increased immobility time in both the TST and FST (Fig. 1E–G). Given the critical role of AHN in depression and cognition, we examined alterations in AHN in both dorsal and ventral dentate gyrus subregions using immunofluorescence combined with BrdU labeling. Ki67, a nuclear protein expressed only in dividing cells, was used as a marker for proliferating neural stem cells (NSCs), while doublecortin (DCX) was employed to label immature neurons. The results revealed a significant reduction in BrdU⁺/DCX⁺ cells in both dorsal and ventral DG of CORT-treated mice (Fig. 1I and S1B). Interestingly, the number of Ki67⁺ cells was significantly increased (Fig. 1H and S1A). In addition, TUNEL assay was used to detect apoptosis in mature neurons; however, no significant difference in TUNEL⁺/NeuN⁺ cells was observed between the control and CORT groups in the DG (Figure S1C and D). These results indicate that CORT administration induces depressive-like behaviors in mice, suppresses neuronal differentiation, and promotes the proliferation of neural stem cells, underscoring a close relationship between depression and altered neurogenesis.

### Impairment of NSCs in the DG suppresses AHN and induces depressive-like behaviors

**Fig. 2:**
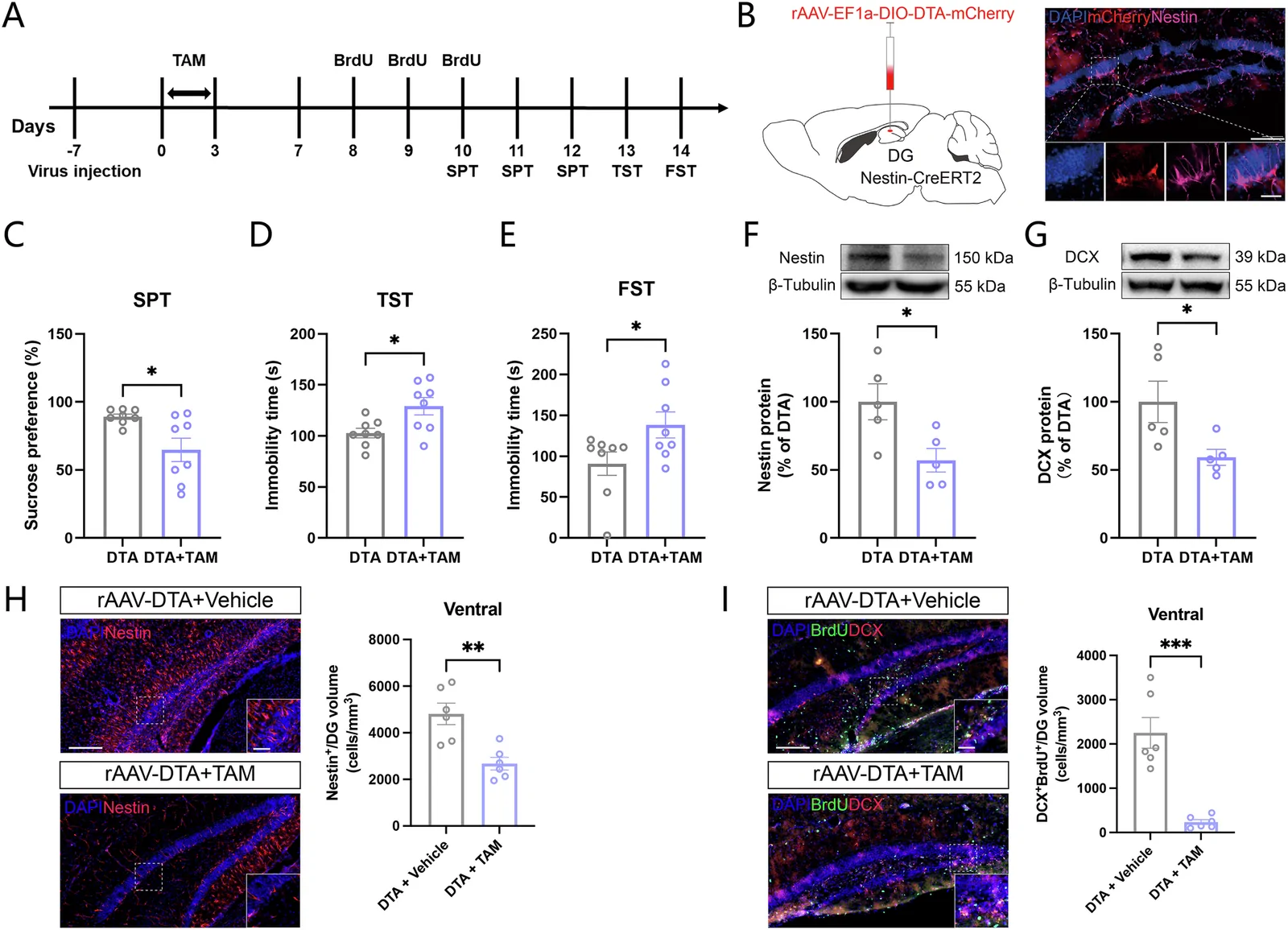
The inhibition of adult hippocampal neurogenesis induces depression-like behaviors in *Nestin*-CreER^T2^ mice. **A** The schedule of the experimental design used to inhibit neuronal differentiation through specific ablation of dentate gyrus neural stem cells and to determine whether this induces depression-like behaviors in mice. TAM: Tamoxifen. **B** Injection of rAAV-DIO-DTA-mCherry into the DG of *Nestin*-CreER^T2^ mice. The fluorescent images show the transfection efficiency of rAAV-DTA-mCherry in hippocampal NSCs. Red: DTA-mCherry; Pink: Nestin. Scale bars: 150 μm for the entire DG and 45 μm for local enlarged panels. **C**–**E** Behavioral performance in SPT (C), TST (D), and FST (E) of *Nestin*-CreER^T2^ mice injected with rAAV-DIO-DTA, assessed 7 days after TAM or vehicle (i.p.) administration. n = 8. **F**, **G** Immunoblots of the Nestin and DCX proteins in the DG after the transfection of the rAAV-DIO-DTA, with β-tubulin as the internal control. The results were normalized against the vehicle group. n = 5. **H**, **I** Representative immunofluorescence images of Nestin^+^ (H) and BrdU^+^/DCX^+^ (I) in the ventral DG of mice with injection of rAAV-DIO-DTA, after TAM or vehicle (i.p.) administration. The density of Nestin^+^ NSCs (H) and BrdU⁺DCX⁺ immature neurons (I) in the ventral DG was quantified as the number of positive cells per mm³. Scale bars: 100 μm for the entire DG and 25 μm for local enlarged panels. n = 6. *P < 0.05; **P < 0.01; ***P < 0.001; error bars represent SEM. Statistical analysis was performed using unpaired t-test.

Given that CORT-induced depression in mice is accompanied by suppressed neurogenesis, we further investigated the critical role of AHN in major depressive disorder by specifically inhibiting neurogenesis in the DG of adult mice and evaluating subsequent depressive-like behaviors. Six-week-old transgenic *Nestin*-CreER^T2^ mice received microinjections of an adeno-associated virus encoding diphtheria toxin A (DTA) into the DG. Seven days post-infection, tamoxifen (180 mg/kg) was administered intraperitoneally for three consecutive days to induce DTA expression, selectively ablating Nestin⁺ neural stem cells and their progeny in the DG (Fig. 2A and B). DTA-mediated ablation resulted in significant depressive-like behaviors in the SPT, TST, and FST, demonstrating that impaired AHN is a key factor in the emergence of depressive phenotypes (Fig. 2C–E). Consistent with this, Nestin protein levels were significantly reduced (Fig. 2F and H), and a marked decrease in DCX protein expression and DCX⁺/BrdU⁺ immature neurons was observed (Fig. 2G and I). Notably, DTA-mediated ablation did not significantly alter Nestin expression or BrdU⁺/DCX⁺ immature neuron density in the dorsal DG (Supplementary Figure S2), indicating that the neurogenic deficit and associated depressive-like behaviors were specifically attributable to impaired AHN in the ventral DG. However, the precise mechanism through which CORT suppresses AHN and leads to depressive-like behaviors remains unclear.

### Aberrant dynorphin expression and reduced levels of neurogenic transcription factors Pax6, Neurog2 and NeuroD1 in the DG

**Fig. 3:**
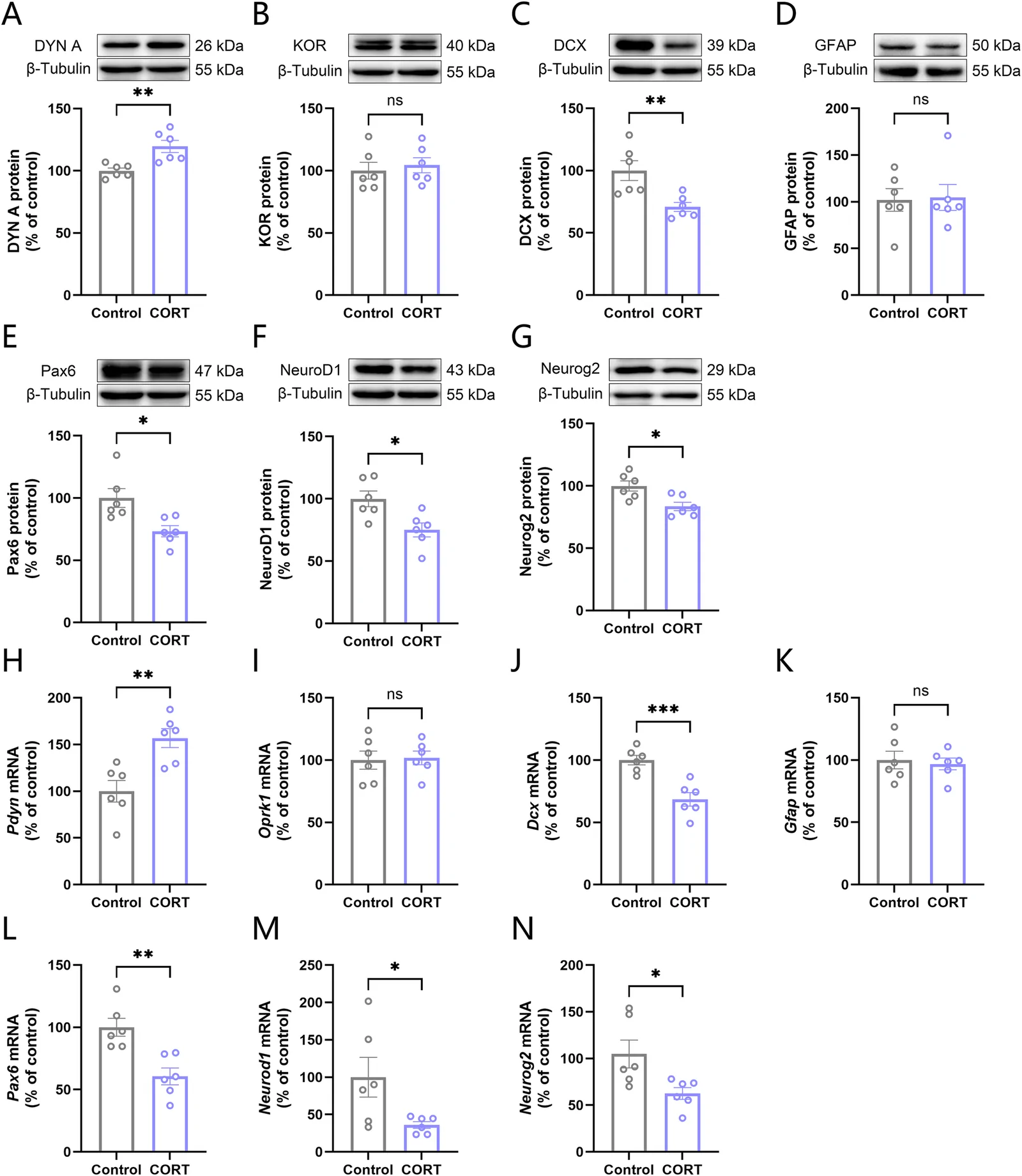
The aberrant expression of dynorphin and neurogenic transcription factor of Pax6, Neurog2 and NeuroD1 in the dentate gyrus of CORT-treated mice. **A**–**G** The representative images of DYN A (A), KOR (B), DCX (C), GFAP (D), Pax6 (E), NeuroD1 (F) and Neurog2 (G) proteins in the DG of mice, detected by Western Blot following behavioral tests (top panels), with their quantifications (bottom panels). The level of β-Tubulin protein was used as the internal control. **H**–**N** The mRNA levels of *Pdyn* (H), *Oprk1* (I), *Dcx* (J), *Gfap* (K), *Pax6* (L), *Neurod1* (M), *Neurog2* (N) in the DG of mice following behavioral tests, detected by qRT-PCR. The results were normalized against the control group. *P < 0.05; **P < 0.01; ***P < 0.001; error bars represent SEM; n = 6. ns: no significance. Statistical analysis was performed using unpaired t-test.

We next examined the expression of the dynorphin/KOR system and neurogenic transcription factors in the DG of CORT-treated mice. The western blot assay and qRT-PCR analyses revealed that dynorphin A protein and its precursor Pdyn mRNA levels were significantly elevated in CORT-treated mice (Fig. 3A and H), suggesting aberrant activation of the dynorphin A/KOR signaling pathway following chronic CORT exposure. In contrast, KOR expression showed no significant change at either protein or mRNA levels (Fig. 3B and I). DCX protein and mRNA expression were markedly reduced (Fig. 3C and J), whereas GFAP levels showed no significant difference (Fig. 3D and K), indicating that the suppression of neurogenesis was not accompanied by a shift toward gliogenesis. Importantly, the transcription factor Pax6 was significantly downregulated at both protein and mRNA levels (Fig. 3E and L), accompanied by decreased expression of its downstream targets NeuroD1 (Fig. 3F and M) and Neurog2 (Fig. 3G and N). These findings underscore the important role of the dynorphin/KOR system along with Pax6, Neurog2, and NeuroD1 in mediating CORT-induced impairment of AHN.

### Regulation of AHN and depressive-like behaviors by the dynorphin/KOR system

**Fig. 4:**
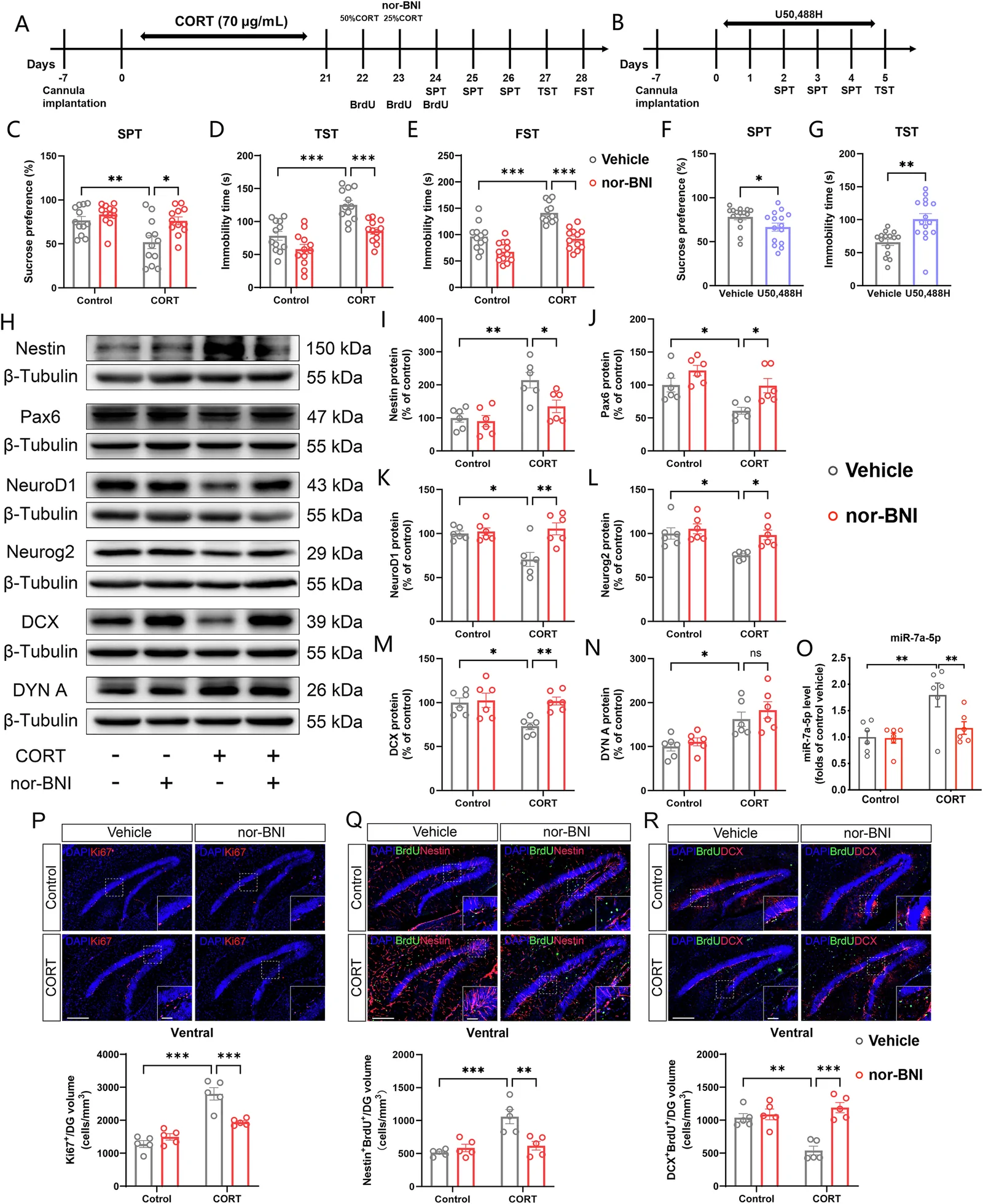
Upregulation of dynorphin and activation of KOR in the dentate gyrus of CORT-treated mice mediated the inhibition of neuronal differentiation and induced depression-like behaviors. **A**, **B** Schematic of the pharmacological experimental protocol used to assess the involvement of the KOR in CORT-induced depression and inhibition of neurogenesis. **C**–**E** The performance of mice with CORT or control in SPT (C), TST (D) and FST (E) after intracranial microinjection of nor-BNI on the day 23 of the experiment. n = 12. **F**, **G** The performance of mice in SPT (F) and TST (G), with intracranial microinjection of U50,488H for 6 consecutive days. n = 15. **H** Representative immunoblots of Nestin, Pax6, NeuroD1, Neurog2, DCX, and DYN A in the DG of mice injected nor-BNI or vehicle, obtained by western blot. **I**–**N** The protein levels of Nestin (I), Pax6 (J), NeuroD1 (K), Neurog2 (L), DCX (M), and DYN A (N) in the hippocampal DG of mice measured by western blot after behavioral testing, with β-Tubulin as the internal control. The results were normalized against the control group. ns: no significance; n = 6. **O** qRT-PCR analysis of miR-7a-5p expression in the DG, with or without CORT and nor-BNI administration. U6 snRNA served as the internal reference. Data are presented as folds of the control + vehicle group. n = 6. **P**–**R** Representative immunofluorescence images and corresponding quantification of Ki67⁺ proliferative neural stem cells (P), BrdU⁺/Nestin⁺ neural stem cells (Q), and BrdU⁺/DCX⁺ immature neurons (R) in the ventral DG of mice treated with nor-BNI or vehicle. Quantification represents the number of positive cells per mm³ of DG volume. Scale bars: 100 μm for the entire DG and 25 μm for local enlarged panels. n = 5. *P < 0.05; **P < 0.01; ***P < 0.001; error bars represent SEM. Statistical analysis was performed using two-way ANOVA in C-E and I-R, and unpaired t-test in F and G.

Given the well-documented role of the dynorphin/KOR system in depression, we sought to investigate whether this system contributes to CORT-induced depressive-like behaviors and suppression of AHN. Using a pharmacological approach, guide cannulas were implanted into the DG seven days prior to CORT administration. During the 25% dose reduction phase of CORT withdrawal, the KOR antagonist nor-BNI (10 μg/0.5 μL per side) was bilaterally microinfused (Fig. 4A). The results showed that nor-BNI significantly alleviated CORT-induced depressive-like behaviors (Fig. 4C–E). Previous studies have demonstrated that KOR agonists can induce depressive-like behaviors in both humans and rodents. To further establish the involvement of the dynorphin/KOR system within the DG in depression, we bilaterally microinjected the KOR agonist U50,488H and evaluated its effects on depressive-like behaviors (Fig. 4B). Continuous administration of U50,488H (10 μg/0.5 μL per side) for six days significantly reduced sucrose preference in the SPT and increased immobility time in the TST, indicating that overactivation of KOR in the DG is sufficient to elicit depressive-like phenotypes (Fig. 4F and G). Histological examination of Nissl-stained coronal sections confirmed that all cannula tips were correctly positioned within or immediately adjacent to the DG for both nor-BNI and U50,488H groups (Supplementary Figure S3). These findings confirm the critical role of the dynorphin/KOR system in the pathogenesis of depression.

In vitro studies have shown that excessive activation of KOR in neural stem cells inhibits neuronal differentiation. Based on our observation that CORT suppresses AHN along with reduced expression of Pax6, Neurog2, and NeuroD1, we further investigated whether the dynorphin/KOR system regulates CORT-induced depressive-like behaviors by modulating AHN. Western blot analysis revealed that nor-BNI significantly reduced Nestin protein levels and restored DCX expression in the DG of CORT-treated mice (Fig. 4I and M). Furthermore, nor-BNI administration also rescued the protein expression of Pax6, Neurog2, and NeuroD1 (Fig. 4J–L). Notably, local infusion of nor-BNI did not affect dynorphin A expression levels (Fig. 4N). Consistent with our previous finding that miR-7a-5p directly targets Pax6 mRNA for translational suppression [25], qRT-PCR analysis revealed that CORT significantly upregulated miR-7a-5p levels in the DG, and this increase was reversed by nor-BNI treatment (Fig. 4O). More directly, nor-BNI treatment decreased the number of Ki67⁺ and Nestin⁺BrdU⁺ cells (Fig. 4P, Q and Figure S4A, C), while increasing DCX⁺BrdU⁺ cells in both dorsal and ventral DG (Fig. 4R and S4B), indicating reduced neural stem cell proliferation and promoted neuronal differentiation. Collectively, these results demonstrate that KOR activity in the DG is closely linked to AHN in CORT-induced depression, and that KOR-mediated regulation of Pax6, Neurog2, and NeuroD1 may underlie the mechanisms by which CORT elicits depressive behaviors.

To further investigate whether the increased Ki67⁺ cells in the DG of CORT-treated mice (Fig. 1H) reflect an expansion of the total NSC pool or enhanced activation of quiescent stem cells, we performed Sox2/Ki67 co-labeling. The total number of Sox2⁺ cells remained comparable across all groups in both the dorsal and ventral DG (Supplementary Figure S5C and F), indicating that the stem cell pool size was not altered by CORT treatment. In contrast, the number of Sox2⁺Ki67⁺ double-positive cells was significantly increased in the CORT group, and this increase was reversed by nor-BNI in both the dorsal and ventral DG (Supplementary Figure S5B and E). These results demonstrate that chronic CORT activates quiescent NSCs into the cell cycle through a KOR-dependent mechanism, without expanding the stem cell pool, and that the resulting progeny accumulate at the Nestin⁺ progenitor stage due to impaired differentiation.

To determine whether KOR signaling acts specifically on Nestin⁺ neural stem/progenitor cells to mediate CORT-induced depressive-like behaviors, we performed conditional deletion of KOR in NSCs by injecting rAAV-*Nestin*-Cre into the DG of *Oprk1*^*LoxP/LoxP*^ mice 14 days before CORT administration (Supplementary Figure S6A and B). Behavioral testing revealed that CORT significantly reduced sucrose preference in AAV-Control mice, and this reduction was significantly attenuated by NSC-specific KOR deletion (Supplementary Figure S6C). Similarly, CORT significantly increased immobility time in the TST in AAV-Control mice, and this increase was significantly attenuated in mice with NSC-specific KOR deletion (Supplementary Figure S6D). At the molecular level, Western blot analysis confirmed that KOR protein levels in the DG were significantly reduced in AAV-*Nes*-Cre groups regardless of CORT treatment, validating the efficiency of NSC-specific KOR deletion (Supplementary Figure S6E and H). Importantly, the CORT-induced downregulation of Pax6 and DCX was significantly rescued by NSC-specific KOR deletion (Supplementary Figure S6F and G). These findings, together with our previous demonstration that KOR directly suppresses the Pax6/Neurog2/NeuroD1 cascade in purified hippocampal NSC cultures via a miR-7a-dependent mechanism [25], provide converging evidence that KOR activation on Nestin⁺ cells is a major contributor to CORT-induced AHN impairment and depressive-like behaviors.

### Effects of Pax6 overexpression in NSCs of the DG on AHN and depressive-like behaviors

**Fig. 5:**
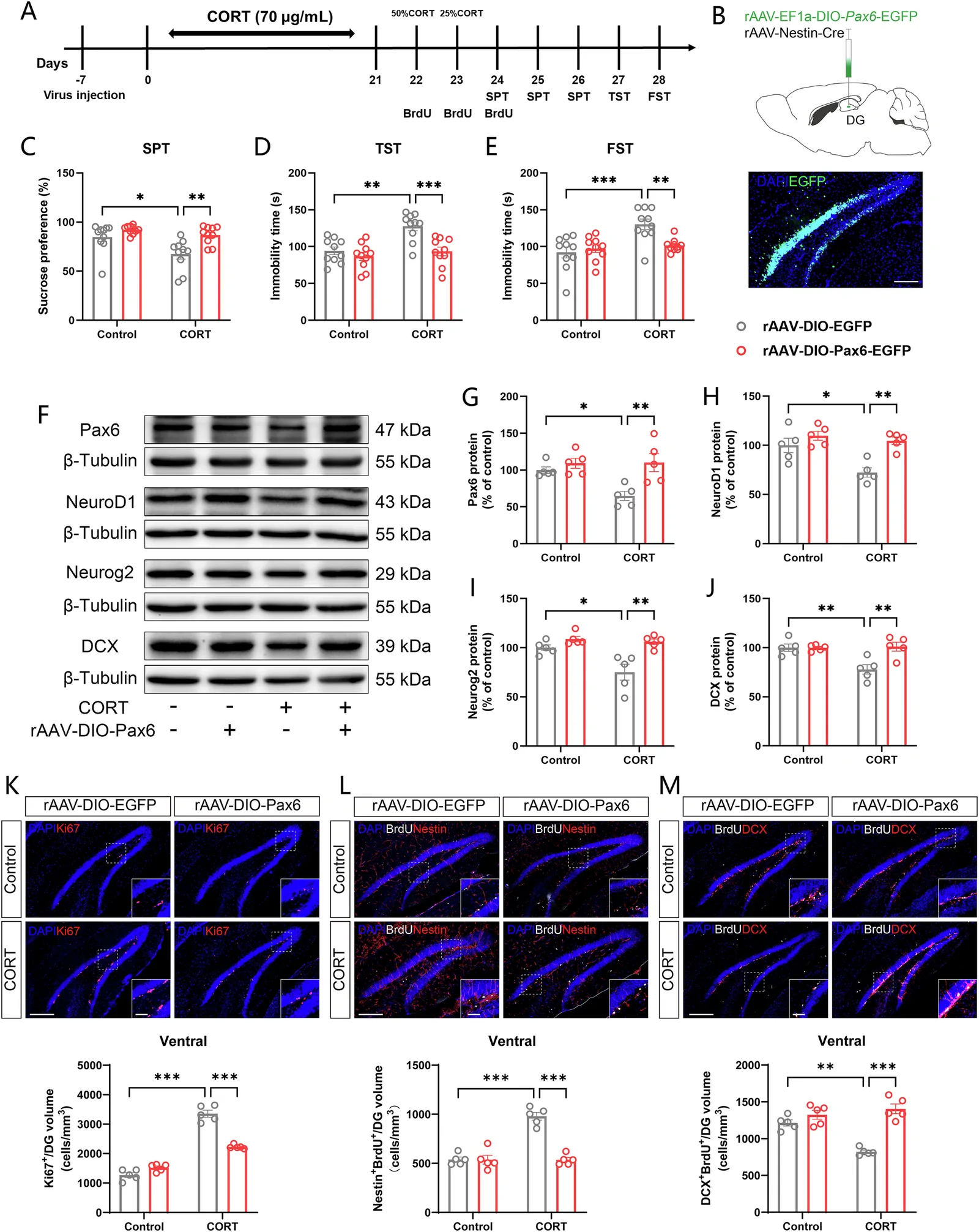
The expression of Pax6 was associated with depression-like behaviors and AHN in mice with CORT treatment. **A** The timeline of the experimental design to determine the effect of rAAV-DIO-*Pax6*-EGFP and rAAV-*Nestin*-Cre transfection on depression-like behaviors and AHN of mice with CORT or control treatment. **B** Schematic representation of viral injections and representative fluorescence image showing co-expression of rAAV-DIO-*Pax6*-EGFP and rAAV-*Nestin*-Cre in the DG. Scale bars: 100 μm for the entire DG. **C**–**E** The performance of mice with CORT or control treatment in SPT (C), TST (D) and FST (E), following co-injection of rAAV-*Nestin*-Cre and rAAV-DIO-*Pax6*-EGFP at the DG. **F** Immunoblots of Pax6, NeuroD1, Neurog2 and DCX in the DG of mice injected with AAV virus, obtained by western blot. **G**–**J** The protein levels of Pax6 (G), NeuroD1 (H), Neurog2 (I), DCX (J) in the DG of mice with CORT or control treatments, measured by western blot after the co-injection of rAAV-DIO-*Pax6*-EGFP and rAAV-*Nestin*-Cre, with β-Tubulin as the internal control. The results were normalized against the control group. **K**–**M** Representative immunofluorescence images and corresponding quantification of Ki67⁺ proliferative neural stem cells (K), BrdU⁺/Nestin⁺ newborn neural stem cells (L), and BrdU⁺/DCX⁺ immature neurons (M) in the ventral DG of mice after the co-injection of rAAV-DIO-*Pax6*-EGFP and rAAV-*Nestin*-Cre. Quantification data represent the number of positive cells per mm³ of DG volume. Scale bars: 100 μm for the entire DG and 25 μm for local enlarged panels. *P < 0.05; **P < 0.01; ***P < 0.001; error bars represent SEM; n = 10 for behavioral tests, and n = 5 for other tests. Statistical analysis was performed using two-way ANOVA.

Having established that Pax6, Neurog2, and NeuroD1 are regulated by the dynorphin/KOR system, and based on previous evidence linking Pax6 to AHN, we further investigated whether the dynorphin/KOR system influences CORT-induced depressive-like behaviors by modulating AHN through Pax6. Given that Pax6 is specifically expressed in NSCs and early progenitor stages, we microinjected recombinant adeno-associated viruses rAAV-*Nestin*-Cre and rAAV-DIO-*Pax6*-EGFP into the DG of wild-type mice seven days before CORT administration to achieve NSC-specific overexpression of Pax6 (Fig. 5A). EGFP fluorescence was observed exclusively in cells within the subgranular zone (Fig. 5B), confirming that *Nestin*-Cre-mediated inversion of the DIO cassette occurred selectively in Nestin⁺ neural stem/progenitor cells. Subsequent behavioral tests were conducted to evaluate the role of Pax6 in CORT-induced depressive-like behaviors. Overexpression of Pax6 in the dentate gyrus NSCs (Fig. 5B and G) significantly ameliorated CORT-induced depressive-like phenotypes in the SPT, TST, and FST (Fig. 5C–E), indicating that Pax6 is a critical factor in mediating CORT-induced depressive behaviors.

After confirming the close relationship between AHN and depression, and the key role of Pax6 in CORT-induced depressive behaviors, we further analyzed the function of Pax6 in AHN under CORT exposure. Western blot analysis showed that Pax6 overexpression in NSCs restored the protein expression of Neurog2, NeuroD1, and DCX, which were suppressed by CORT (Fig. 5F–J). Furthermore, immunofluorescence and BrdU labeling revealed that Pax6 overexpression reduced the number of Ki67⁺ cells and Nestin⁺BrdU⁺ cells (Fig. 5K and L and supplementary Figure S7A and B), while increasing the number of DCX⁺BrdU⁺ cells in both dorsal and ventral DG (Fig. 5M and S7C), suggesting that Pax6 attenuates CORT-induced NSC proliferation and promotes neuronal differentiation. Taken together, these results demonstrate that CORT suppresses AHN and induces depressive-like behaviors at least partially through downregulation of Pax6 in NSCs.

### Downregulation of Pax6 in NSCs suppresses AHN and promotes depressive-like behaviors

**Fig. 6:**
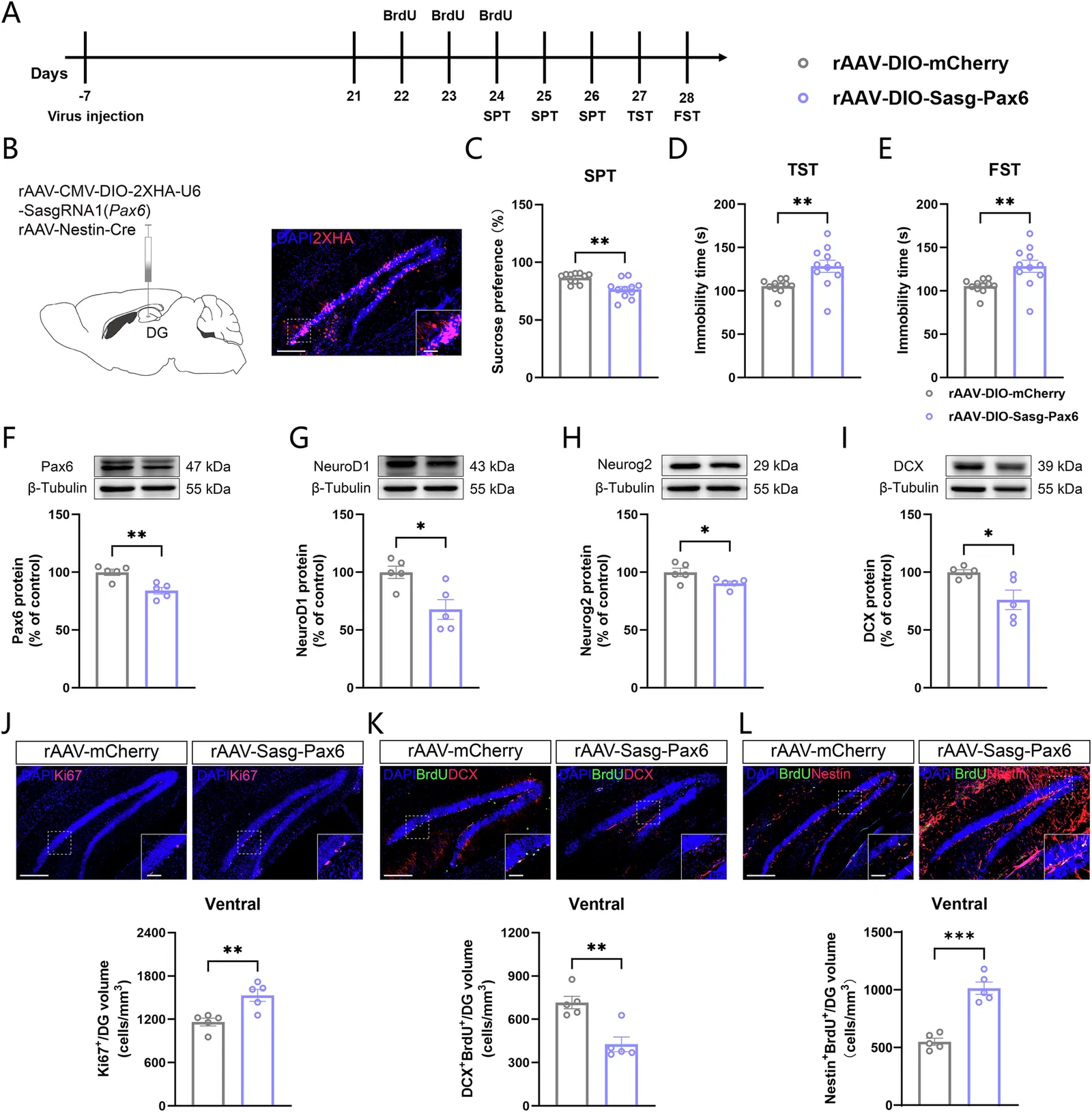
Suppression of Pax6 expression in the DG underlies the deficits in AHN and the emergence of depression-like behaviors. **A** The timeline of the experimental design to determine the effect of rAAV-DIO-SasgRNA1(Pax6) and rAAV-*Nestin*-Cre transfection on depression-like behaviors and AHN of mice. **B** Schematic representation of viral injections. The fluorescence image showing co-expression of rAAV-DIO-SasgRNA1(*Pax6*) and rAAV-*Nestin*-Cre in the DG. Scale bars: 100 μm for the entire DG. **C**–**E** The performance of mice with CORT or control treatment in SPT, TST and FST, following co-injection of rAAV-DIO-SasgRNA1(Pax6) and rAAV-*Nestin*-Cre into the DG. n = 11 per group. **F**–**I** Representative immunoblot images and corresponding protein levels of Pax6 (F), NeuroD1 (G), Neurog2 (H), DCX (I) in the DG of mice were measured by Western Blot after the co-injection of rAAV-DIO-SasgRNA1(*Pax6*) and rAAV-*Nestin*-Cre, with β-tubulin as the internal control. The results were normalized against the control group. **J**–**L** Representative immunofluorescence images and corresponding quantification of Ki67⁺ proliferative neural stem cells (J), BrdU⁺/DCX⁺ immature neurons (K), and BrdU⁺/Nestin⁺ newborn neural stem cells (L) in the ventral DG of mice after the co-injection of rAAV-DIO-SasgRNA1(*Pax6*) and rAAV-*Nestin*-Cre. Quantification data represent the number of positive cells per mm³ of DG volume. Scale bars: 100 μm for the entire DG and 25 μm for local enlarged panels. *P < 0.05; **P < 0.01; ***P < 0.001; error bars represent SEM; n = 11 for behavioral tests, and n = 5 for other tests. Statistical analysis was performed using unpaired t-test.

Given that suppression of Pax6 contributes to impaired AHN and depressive-like behaviors in CORT-treated mice, we further investigated whether Pax6 represents a key mediator in the development of depression. To specifically downregulate Pax6 expression in NSCs, wild-type mice received microinjections of rAAV-*Nestin*-Cre and rAAV-DIO-2×HA-U6-sgRNA(*Pax6*) into the DG (Fig. 6A), leading to effective Pax6 knockdown after 4 weeks of viral expression (Fig. 6B and F). HA fluorescence was observed exclusively in cells within the subgranular zone (Fig. 6B), confirming that *Nestin*-Cre-mediated inversion of the DIO cassette occurred selectively in Nestin⁺ neural stem/progenitor cells. Behavioral tests revealed that mice with NSC-specific *Pax6* knockdown exhibited significant depressive-like behaviors in the SPT, TST, and FST (Fig. 6C–E).

Given that CORT administration significantly suppressed AHN and downregulated Pax6, we next examined the role of Pax6 in regulating AHN (through Neurog2 and NeuroD1) in the development of depressive-like behaviors. Western blot analysis showed that reduced Pax6 expression in the DG resulted in significantly decreased protein levels of Neurog2, NeuroD1, and DCX (Fig. 6F–I). More directly, immunofluorescence and BrdU labeling demonstrated that Pax6 knockdown led to a marked reduction in DCX⁺BrdU⁺ cells (Fig. 6K and S8B), along with a significant increase in Ki67⁺ cells and Nestin⁺BrdU⁺ cells in both dorsal and ventral DG compared with control mice (Fig. 6J and L and supplementary Figure S8A and C). These findings indicate that downregulation of Pax6 strongly inhibits neuronal differentiation and promotes the proliferation of neural stem cells. Together, these results demonstrate that Pax6 expression in DG NSCs is essential for maintaining AHN through Neurog2 and NeuroD1 and exerts an antidepressant-like function.

## Discussion

Through a series of in vivo experiments, this study provides evidence for a cellular and molecular pathway through which CORT may contribute to depressive-like behaviors in mice. Our findings indicate that CORT upregulates dynorphin expression in the DG, leading to overactivation of the KOR, which subsequently suppresses the transcription factor Pax6 and its downstream targets Neurog2 and NeuroD1, ultimately impairing AHN. The 21-day CORT model recapitulated core depressive features including anhedonia, behavioral despair, and dysregulated HPA axis feedback, affirming its validity for studying the neurobiological mechanisms of depression. Furthermore, employing *Nestin*-CreER^T2^/DTA-mediated genetic ablation of Nestin⁺ cells, we demonstrated that direct suppression of AHN is sufficient to induce depressive-like behaviors, thereby establishing AHN deficiency as a pivotal contributor to depression rather than merely an epiphenomenon of CORT exposure.

An intriguing observation is that chronic CORT treatment increased Ki67⁺ proliferating cells and Nestin⁺/BrdU⁺ progenitors in the DG (Figs. 1H, S1A, 4I, and Q), while simultaneously reducing DCX⁺/BrdU⁺ immature neurons (Figs. 1I, S1B, 4M, and R). This pattern contrasts with psychosocial stress models such as CUMS or CSDS, which typically report suppressed NSC proliferation [34, 35]. The discrepancy likely reflects the distinct nature of chronic exogenous CORT administration, which produces sustained supraphysiological glucocorticoid levels that may preferentially disrupt differentiation-promoting transcriptional programs, such as the Pax6/Neurog2/NeuroD1 cascade identified here, while leaving intact, or even stimulating, the proliferative machinery of NSCs. Our data suggest that the surplus of proliferating cells accumulates at the Nestin⁺ progenitor stage, representing a “differentiation bottleneck” rather than a generalized enhancement of neurogenesis. The unchanged GFAP levels (Fig. 3D and K) argue against a shift toward astrocytic differentiation, and the absence of increased TUNEL⁺/NeuN⁺ cells (Figure S1C and D) indicates that mature neuron apoptosis is not a major contributing factor. However, we cannot exclude the possibility that some progenitors are lost through non-apoptotic pathways or that a minor contribution from alternative lineage differentiation (e.g., oligodendrocyte precursors) occurs, which warrants future investigation. This model also reconciles the seemingly paradoxical finding that both DTA-mediated depletion (Fig. 2) and CORT-induced expansion (Fig. 4) of the Nestin⁺ pool are associated with depressive-like behaviors: in both scenarios, the critical variable is the output of functional new neurons, not the size of the progenitor pool per se. DTA ablation directly removes the source of new neurons, while CORT-induced Pax6 suppression arrests progenitors in a proliferative but non-productive state. Both conditions converge on a net reduction in functional neurogenesis, consistent with the concept that AHN homeostasis is essential for normal mood regulation. Whether the KOR/Pax6 axis is similarly dysregulated in CUMS or CSDS models remains an important question for future investigation.

Pharmacological experiments provided evidence that both CORT-induced depressive-like phenotypes and AHN suppression are largely mediated by excessive activation of the dynorphin/KOR system in the DG: intra-DG infusion of nor-BNI reversed depressive-like behaviors and rescued impaired neurogenesis, while direct KOR activation with U50,488H recapitulated these effects. A key question, however, is whether KOR acts specifically on Nestin⁺ NSCs or through broader modulation of other KOR-expressing cell types. Our conditional deletion of KOR in Nestin⁺ cells (Supplementary Figure S6) provides direct genetic evidence that KOR signaling within the NSC lineage is a major contributor to CORT-induced neurogenic impairment and depressive phenotypes, complementing our earlier in vitro finding that KOR directly suppresses the Pax6/Neurog2/NeuroD1 cascade in hippocampal NSCs [25]. However, the partial, rather than complete, behavioral rescue implies that other KOR-expressing populations likely contribute. KOR is expressed on mature granule neurons and GABAergic interneuron subtypes including parvalbumin-positive and somatostatin-positive neurons [36, 37], and modulation of these populations could alter the excitatory-inhibitory balance within the DG microcircuit. In a broader context, the dynorphin/KOR system has emerged as a critical modulator of emotional states across multiple brain regions: KOR activation in the VTA-NAc pathway mediates stress-induced aversion and anhedonia [38], while KOR signaling in the amygdala contributes to anxiety-related behaviors through modulation of CRF-containing neurons [39]. Our findings add a new dimension by revealing that KOR activation in the hippocampal DG suppresses adult neurogenesis through Pax6 downregulation, positioning the hippocampal KOR/Pax6/neurogenesis axis as a complementary mechanism to reward circuit dysfunction in depression. The antidepressant potential of KOR antagonism, including aticaprant, currently in clinical trials for MDD [40], may thus arise from the convergence of multiple parallel mechanisms: restoration of Pax6-dependent neurogenesis, circuit-level normalization of DG network activity, and modulation of mesolimbic reward processing.

Our previous in vitro study demonstrated that KOR activation regulates Pax6 expression through modulation of miR-7a-5p maturation in hippocampal NSCs [25]. KOR is a Gi/o-coupled receptor whose activation engages multiple downstream kinase cascades, including the MAPK/ERK pathway [18]. Given that ERK signaling regulates the processing of specific miRNAs through phosphorylation of TRBP [41], it is plausible that CORT-induced dynorphin/KOR activation modulates p-ERK activity, which in turn enhances miR-7a-5p maturation and suppresses Pax6 translation. Indeed, our data showing that CORT upregulates miR-7a-5p in the DG and that this effect is reversed by nor-BNI (Fig. 4O) provide in vivo support for this model. However, because ERK phosphorylation is a rapid and transient event, steady-state p-ERK levels after 21 days of chronic CORT may not accurately reflect the dynamic signaling events that drive cumulative miR-7a-5p upregulation. Characterizing the temporal dynamics of KOR/ERK/miR-7a-5p signaling using acute KOR activation paradigms represents an important future direction. A related observation is that Pax6 overexpression via AAV did not produce a statistically significant increase in Pax6 protein in the control group (Fig. 5G), despite equivalent Nestin⁺ target cells being available for Cre-mediated recombination. We attribute this to the fact that Pax6 is already expressed at high baseline levels in unstressed NSCs; exogenous Pax6 may produce only a modest increment that fails to reach significance due to a ceiling effect or homeostatic degradation mechanisms. In contrast, in the CORT group, where endogenous Pax6 is markedly suppressed, the exogenous Pax6 effectively compensates for the deficit, producing a pronounced restoration. This is consistent with previous reports that Pax6 controls NSC self-renewal and neurogenesis in a tightly concentration-dependent manner [28]. Furthermore, our Pax6 gain- and loss-of-function experiments (Figs. 5 and 6) establish both the necessity and sufficiency of Pax6 as a key executor downstream of KOR: overexpression of Pax6 in NSCs counteracted CORT-induced deficits, while knockdown of Pax6 phenocopied them, directly linking Pax6 functional status to AHN integrity and depressive-like behaviors. Whether CORT also regulates NSC proliferation through Pax6-Notch pathway interactions [42, 43] warrants further investigation.

Several limitations of the present study should be acknowledged. First, only male mice were used; given sex differences in HPA axis reactivity and hippocampal neurogenesis, future studies should examine whether the KOR/Pax6 axis operates similarly in female animals. Second, the CORT model represents only one aspect of the multifactorial etiology of depression and may not fully recapitulate the heterogeneous neurobiological features of human MDD. Third, although our KOR conditional knockout experiment provides direct genetic evidence for the involvement of KOR in Nestin⁺ cells, the pharmacological experiments using local nor-BNI infusion do not offer cell-type specificity, and the broader circuit-level contributions of KOR on mature neurons and interneurons have not been experimentally dissected. Fourth, the precise in vivo signaling intermediates between KOR activation and Pax6 suppression, including the roles of miR-7a-5p and downstream kinase cascades, have not been directly characterized in the current CORT model. Finally, while our AAV-based manipulations demonstrate the necessity and sufficiency of Pax6 in regulating AHN and mood-related behaviors, the functional integration and long-term survival of newly generated neurons were not directly assessed. Despite these limitations, our findings support a model in which CORT promotes depressive-like behaviors through activation of the dynorphin/KOR system, subsequent suppression of Pax6 and its downstream effectors, and ultimately the disruption of AHN. These findings provide a foundation for developing novel antidepressant strategies targeting KOR or Pax6, and future research should explore the potential of combining KOR antagonists with existing antidepressant drugs.

## Conclusion

Our findings reveal a molecular pathway wherein corticosterone (CORT)-induced upregulation of dynorphin leads to excessive kappa-opioid receptor (KOR) activation in the dentate gyrus, which suppresses the transcription factor Pax6 and its downstream effectors Neurog2 and NeuroD1. This cascade contributes to the impairment of Pax6-dependent adult hippocampal neurogenesis by inhibiting neuronal differentiation and promoting aberrant neural stem cell proliferation, which may underlie depression-like behaviors. These results suggest that the dynorphin/KOR-Pax6 axis represents a mechanism linking stress hormones to neurogenic deficits and depressive phenotypes and point to KOR and Pax6 as promising therapeutic targets for novel antidepressant interventions.

## Supplementary information

Supplementary Table S1

Supplementary Table S2

Supplementary Table S3

Supplementary Figure S1

Supplementary Figure S2

Supplementary Figure S3

Supplementary Figure S4

Supplementary Figure S5

Supplementary Figure S6

Supplementary Figure S7

Supplementary Figure S8

Supplementary Figure Legends

Original Blots

## Data Availability

All relevant raw data that support the findings of the current study are available from the corresponding author upon reasonable request.
