# Supplementary material for: Kappa-opioid receptor activation promotes depression-like behaviors by suppressing Pax6-dependent adult hippocampal neurogenesis
Source: Transl Psychiatry. 2026 Jul 3;16:496. doi: 10.1038/s41398-026-04230-z (PMC13620123; doi:10.1038/s41398-026-04230-z)
Supplement: Supplementary file 1 [file 41398_2026_4230_MOESM12_ESM.docx]

**Supplementary Figure Legends**

**Fig. S1 The differentiation and proliferation of neural stem cells and apoptosis of mature neurons in the DG in mice with CORT treatment.**

(A, B) Representative immunofluorescence images and corresponding quantification of Ki67⁺ proliferative neural stem cells (A) and BrdU⁺/DCX⁺ immature neurons (B) in the dorsal DG of mice with CORT treatment. Quantification data represent the number of positive cells per mm³ of DG volume. n=6.

(C, D) Representative immunofluorescence images showing TUNEL⁺/NeuN⁺ apoptotic mature neurons in the dorsal and ventral DG of CORT-treated mice. Quantification of cell apoptosis by comparing the average fluorescence of TUNEL and NeuN, calculated as the TUNEL/NeuN ratio. Scale bars: 100 μm the entire DG and 25 μm for local enlarged panels. ns: no significance; n≥6.

**P<0.01; ***P<0.001; error bars represent SEM. Statistical analysis was performed using unpaired t-test (A, B) and one-way ANOVA (C, D).

**Fig. S2 Effect of NSCs ablation on AHN in the dorsal DG of Nestin-CreER^T2^ mice.**

Representative immunofluorescence images of Nestin^+^ (A) and BrdU^+^/DCX^+^ (B) in the dorsal DG of Nestin-CreER^T2^ mice with injection of rAAV-DIO-DTA, after TAM or vehicle (i.p.) administration. The density of Nestin^+^ NSCs (A) and BrdU⁺/DCX⁺ immature neurons in dorsal DG was quantified as the number of positive cells per mm³ following behavioral tests (B). Scale bars: 100 μm the entire DG and 25 μm for local enlarged panels. ns: no significance; error bars represent SEM; n=6. Statistical analysis was performed using unpaired t-test.

**Fig. S3 Histological verification of intra-DG cannula placement.**

(A) Representative photomicrographs of Nissl-stained coronal sections showing bilateral guide cannula placement targeting the DG. The internal injection cannula extended 1 mm beyond the tip of the guide cannula to reach the DG. Scale bars: 1 mm. DG, dentate gyrus.

(B) Schematic diagrams of coronal sections (adapted from Paxinos and Franklin Mouse Brain Atlas) at Bregma -2.15 mm and -2.27 mm, showing the verified injection sites for all animals included in the Fig. 4 analyses. Red dots: nor-BNI (n = 36); blue dots, U50,488H (n = 15). Each dot represents one injection site. Animals with misplaced cannulas were excluded from analysis.

**Fig. S4 Upregulation of dynorphin in the DG of CORT-treated mice underlies the impairment of AHN in the dorsal region.**

Immunofluorescence images and corresponding quantification of Ki67⁺ proliferative neural stem cells (A), BrdU⁺/DCX⁺ immature neurons (B), and BrdU⁺/Nestin⁺ newborn neural stem cells (C) in the dorsal DG of mice treated with nor-BNI or vehicle. Quantification data represent the number of positive cells per mm³ of DG volume. Scale bars: 100 μm the entire DG and 25 μm for local enlarged panels. *P<0.05; **P<0.01; ***P<0.001; error bars represent SEM; n=5. Statistical analysis was performed using two-way ANOVA.

**Fig. S5 Sox2/Ki67 co-labeling in the DG of CORT- and nor-BNI-treated mice.**

(A & D) Representative immunofluorescence images of Sox2 (red) and Ki67 (green) co-labeling in the dorsal (A) and ventral DG (D). Blue: DAPI. Scale bars: 100 μm for the overview panels and 25 μm for the locally enlarged panels.

(B & E) Quantification of Sox2⁺Ki67⁺ double-positive cells in the dorsal (B) and ventral (E) DG (two-way ANOVA with Bonferroni's post hoc test; ***P < 0.001; n = 5.

(C & F) Quantification of total Sox2⁺ cells in the dorsal (C) and ventral (F) DG (two-way ANOVA; n = 5).

**P<0.01; ***P<0.001; error bars represent SEM; n=5. Statistical analysis was performed using two-way ANOVA.

**Fig. S6 Conditional deletion of KOR in Nestin⁺ neural stem cells attenuates CORT-induced depressive-like behaviors and restores Pax6 and DCX expression.**

(A) Timeline of the experimental design.

(B) Schematic representation of bilateral viral injection into the DG of *Oprk1^LoxP/LoxP^* mice.

(C, D) Sucrose preference (SPT, C) and immobility time (TST, D) of *Oprk1^LoxP/LoxP^* mice injected with rAAV-Control or rAAV-*Nes*-Cre, with or without CORT treatment. n = 8.

(E) Representative immunoblots of KOR, DCX, Pax6, and Cofilin (loading control) in the DG.

(F–H) Quantification of Pax6 (F), DCX (G), and KOR (H) protein levels in the DG, normalized against Cofilin and expressed as percentage of the AAV-Control + Vehicle group. n = 6.

*P < 0.05; **P < 0.01; ***P < 0.001; error bars represent SEM; Statistical analysis was performed using two-way ANOVA.

**Fig. S7 Effect of Pax6 overexpression on AHN in the dorsal DG of CORT-treated mice.**

Immunofluorescence images and corresponding quantification of Ki67⁺ proliferative neural stem cells (A), BrdU⁺/Nestin⁺ newborn neural stem cells (B), and BrdU⁺/DCX⁺ immature neurons (C) in the dorsal DG of mice after the co-injection of rAAV-DIO-*Pax6* and rAAV-*Nestin*-Cre. Quantification data represent the number of positive cells per mm³ of DG volume. Scale bars: 100 μm the entire DG and 25 μm for local enlarged panels. **P<0.01; ***P<0.001; error bars represent SEM; n=5. Statistical analysis was performed using two-way ANOVA.

**Fig. S8 Downregulation of Pax6 expression in the DG underlies the deficits in dorsal AHN.**

Representative immunofluorescence images and corresponding quantification of Ki67⁺ proliferative neural stem cells (A), BrdU⁺/DCX⁺ immature neurons (B), and BrdU⁺/Nestin⁺ newborn neural stem cells (C) in the dorsal DG of mice after the co-injection of rAAV-DIO-SasgRNA1(*Pax6*) and rAAV-*Nestin*-Cre. Quantification data represent the number of positive cells per mm³ of DG volume. Scale bars: 100 μm the entire DG and 25 μm for local enlarged panels. **P<0.01 compared with control; error bars represent SEM; n=5. Statistical analysis was performed using unpaired t-test.
