# Supplementary material for: Kappa-opioid receptor activation promotes depression-like behaviors by suppressing Pax6-dependent adult hippocampal neurogenesis
Source: Transl Psychiatry. 2026 Jul 3;16:496. doi: 10.1038/s41398-026-04230-z (PMC13620123; doi:10.1038/s41398-026-04230-z)
Supplement: Supplementary file 3 [file 41398_2026_4230_MOESM1_ESM.docx]

| **Primers** | **Sequences** |
| --- | --- |
| m*Actin* forward | 5’-AGAGGGAAATCGTGCGTGACATC-3’ |
| m*Actin* reverse | 5’-ACCGCTCGTTGCCAATAGTGATG-3’ |
| m*Dcx* forward | 5’-GGGAAAGCACAAGGCACACG-3’ |
| m*Dcx* reverse | 5’-GGTGGAACCACAGCAACTTTTCC-3’ |
| m*Gfap* forward | 5’-TCCTGGAACAGCAAAACAAG-3’ |
| m*Gfap* reverse | 5’-CAGCCTCAGGTTGGTTTCAT-3’ |
| m*Pax6* forward | 5’-CGGTGAGCAGATGTGTGAGA-3’ |
| m*Pax6* reverse | 5’-TCACTCCGCTGTGACTGTTC-3’ |
| m*Neurog2* forward | 5’-AGAGGTGGCCCTTGCAATC-3’ |
| m*Neurog2* reverse | 5’-CACACGCCATAGTCCTCTTTGA-3’ |
| m*Neurod1* forward | 5’-CTGATCTGGTCTCCTTCGTACAG-3’ |
| m*Neurod1* reverse | 5’-GGTTCTGCTCAGGCAAGAAAG-3’ |
| m*Oprk1* forward | 5’-CACCAAAGTCAGGGAAGATGT-3’ |
| m*Oprk1* reverse | 5’-GCAGACAATGATGATGAGGAC-3’ |
| m*Pdyn* forward | 5’-TTTGGCAACGGAAAAGAATC-3’ |
| m*Pdyn* reverse | 5’-CATCTCGGAACTCCTCTTGG-3’ |
| mmu-miR-7a-5p | 5’-TGGAAGACTAGTGATTTTGT-3’ |
