# Supplementary material for: Kappa-opioid receptor activation promotes depression-like behaviors by suppressing Pax6-dependent adult hippocampal neurogenesis
Source: Transl Psychiatry. 2026 Jul 3;16:496. doi: 10.1038/s41398-026-04230-z (PMC13620123; doi:10.1038/s41398-026-04230-z)
Supplement: Supplementary file 4 [file 41398_2026_4230_MOESM2_ESM.docx]

**Table S2	Antibodies**

**Primary Antibodies**

**Immunoblotting**

| Antibodies | Conditions |
| --- | --- |
| Anti-Pax6 [ab109233] (Abcam) | Rabbit monoclonal IgG, 1:1,000 |
| Anti-Neurog2 [bs-3692R] (Bioss) | Rabbit Polyclonal IgG, 1:1,000 |
| Anti-NeuroD1 [ab213725] (Abcam) | Rabbit monoclonal IgG, 1:1,000 |
| Anti-GFAP [ab4674] (Abcam) | Rabbit monoclonal IgG, 1:1,000 |
| Anti-Doublecortin [ab18723] (Abcam) | Rabbit Polyclonal IgG, 1:1,000 |
| Anti-Nestin [ab221660] (Abcam) | Rabbit monoclonal IgG, 1:1,000 |
| Anti-Dynorphin A [ab82509] (Abcam) | Rabbit monoclonal IgG, 1:1,000 |
| Anti-KOR [A14035] (ABclonal) | Rabbit monoclonal IgG, 1:1,000 |
| Anti-β-Tubulin [2146S] (CST) | Rabbit monoclonal IgG, 1:1,000 |
| Anti-Cofilin [ab54532] (Abcam) | Mouse monoclonal IgG, 1:1,000 |

**Immunocytochemistry**

| Antibodies | Conditions |
| --- | --- |
| Anti-BrdU [ab6326] (Abcam) | Rat monoclonal IgG, 1:200 |
| Anti-Nestin [ab221660] (Abcam) | Rabbit monoclonal IgG, 1:400 |
| Anti-Doublecortin[ab18723] (Abcam) | Rabbit polyclonal IgG, 1:1,000 |
| Anti-Ki67 [ab15580] (Abcam) | Rabbit monoclonal IgG, 1:500 |
| Anti-HA-Tag [3724T] (CST) | Rabbit monoclonal IgG, 1:700 |
| Anti-Sox2 [ab79351] (Abcam) | Mouse monoclonal IgG1, 1:200 |

**Secondary Antibodies**

**Immunoblotting**

| Antibodies | Conditions |
| --- | --- |
| Anti-rabbit IgG, HRP-linked Antibody [7074] (CST) | Goat IgG, 1:2,000 |
| Anti-mouse IgG, HRP-linked Antibody [7076] (CST) | Horse IgG, 1:2,000 |

**Immunohistochemistry**

| Antibodies (ThermoFisher) | Conditions |
| --- | --- |
| Alexa Fluor® plus 488 goat anti-rat IgG | Goat polyclonal IgG, 1:1,000 |
| Alexa Fluor® plus 594 goat anti-rat IgG | Goat polyclonal IgG, 1:1,000 |
| Alexa Fluor® plus 594 goat anti-rabbit IgG | Goat polyclonal IgG, 1:1,000 |
| Alexa Fluor® plus 647 goat anti-mouse IgG | Goat polyclonal IgG, 1:1,000 |
| Alexa Fluor® plus 488 goat anti-rabbit IgG | Goat polyclonal IgG, 1:1,000 |
