# Supplementary material for: Kappa-opioid receptor activation promotes depression-like behaviors by suppressing Pax6-dependent adult hippocampal neurogenesis
Source: Transl Psychiatry. 2026 Jul 3;16:496. doi: 10.1038/s41398-026-04230-z (PMC13620123; doi:10.1038/s41398-026-04230-z)
Supplement: Supplementary file 5 [file 41398_2026_4230_MOESM3_ESM.docx]

**Supplementary Table S3** Detailed statistical results for all comparisons in this study.

| **Figure** | **Comparison** | **Test** | **F/t** | **P Value** |
| --- | --- | --- | --- | --- |
| 1B | Control vs. CORT | Two-way ANOVA | F (7, 152) = 3.867 | 0.0007 |
| 1C | Control vs. CORT | Two-way ANOVA | F (6, 56) = 6.237 | < 0.0001 |
| 1D | Control vs. CORT | unpaired t-test | t (10) = 4.757 | 0.0008 |
| 1E | Control vs. CORT | unpaired t-test | t (20) = 2.199 | 0.0398 |
| 1F | Control vs. CORT | unpaired t-test | t (20) = 4.087 | 0.0006 |
| 1G | Control vs. CORT | unpaired t-test | t (20) = 4.219 | 0.0004 |
| 1H | Control vs. CORT | unpaired t-test | t (20) = 5.050 | 0.0005 |
| 1I | Control vs. CORT | unpaired t-test | t (10) = 3.986 | 0.0026 |
| 2C | DTA + Vehicle vs. DTA +TAM | unpaired t-test | t (14) = 2.796 | 0.0143 |
| 2D | DTA + Vehicle vs. DTA +TAM | unpaired t-test | t (14) = 2.733 | 0.0162 |
| 2E | DTA + Vehicle vs. DTA +TAM | unpaired t-test | t (14)=2.206 | 0.0446 |
| 2F | DTA + Vehicle vs. DTA +TAM | unpaired t-test | t (8)=2.730 | 0.0259 |
| 2G | DTA + Vehicle vs. DTA +TAM | unpaired t-test | t (8)=2.510 | 0.0364 |
| 2H | DTA + Vehicle vs. DTA +TAM | unpaired t-test | t (10) = 4.006 | 0.0025 |
| 2I | DTA + Vehicle vs. DTA +TAM | unpaired t-test | t (10) = 5.750 | 0.0002 |
| 3A | Control vs. CORT | unpaired t-test | t (10) = 3.654 | 0.0044 |
| 3B | Control vs. CORT | unpaired t-test | t (10) = 0.4918 | 0.6335 |
| 3C | Control vs. CORT | unpaired t-test | t (10) = 3.367 | 0.0072 |
| 3D | Control vs. CORT | unpaired t-test | t (10) = 0.1423 | 0.8897 |
| 3E | Control vs. CORT | unpaired t-test | t (10) = 3.032 | 0.0126 |
| 3F | Control vs. CORT | unpaired t-test | t (10) = 2.970 | 0.0141 |
| 3G | Control vs. CORT | unpaired t-test | t (10) = 3.095 | 0.0113 |
| 3H | Control vs. CORT | unpaired t-test | t (10) = 3.692 | 0.0042 |
| 3I | Control vs. CORT | unpaired t-test | t (10) = 0.1930 | 0.8508 |
| 3J | Control vs. CORT | unpaired t-test | t (10) = 4.737 | 0.0008 |
| 3K | Control vs. CORT | unpaired t-test | t (10) = 0.3715 | 0.718 |
| 3L | Control vs. CORT | unpaired t-test | t (10) = 4.001 | 0.0025 |
| 3M | Control vs. CORT | unpaired t-test | t (10) = 2.367 | 0.0394 |
| 3N | Control vs. CORT | unpaired t-test | t (10) = 2.617 | 0.0257 |
| 4C | Control + Vehicle vs. CORT +Vehicle  CORT + Vehicle vs. CORT + nor-BNI | Two-way ANOVA | F (1, 44) = 9.081  t (44) = 3.381  t (44) = 3.326 | 0.0091  0.0106 |
| 4D | Control + Vehicle vs. CORT +Vehicle  CORT + Vehicle vs. CORT + nor-BNI | Two-way ANOVA | F (1, 44) = 21.53  t (44) = 5.118  t (44) = 4.338 | < 0.0001  0.0005 |
| 4E | Control + Vehicle vs. CORT +Vehicle  CORT + Vehicle vs. CORT + nor-BNI | Two-way ANOVA | F (1, 44) = 42.06  t (44) = 5.244  t (44) = 5.764 | < 0.0001  < 0.0001 |
| 4F | Vehicle vs. U50,488H | unpaired t-test | t (28) = 2.055 | 0.0483 |
| 4G | Vehicle vs. U50,488H | unpaired t-test | t (28) = 3.626 | 0.0011 |
| 4I | Control + Vehicle vs. CORT +Vehicle  CORT + Vehicle vs. CORT + nor-BNI | Two-way ANOVA | F (1, 20) = 3.582  t (20) = 4.387  t (20) = 3.038 | 0.0017  0.0383 |
| 4J | Control + Vehicle vs. CORT +Vehicle  CORT + Vehicle vs. CORT + nor-BNI | Two-way ANOVA | F (1, 20) = 0.8106  t (20) = 3.108  t (20) = 3.035 | 0.0328  0.0386 |
| 4K | Control + Vehicle vs. CORT +Vehicle  CORT + Vehicle vs. CORT + nor-BNI | Two-way ANOVA | F (1, 20) = 7.774  t (20) = 3.553  t (20) = 4.207 | 0.0119  0.0026 |
| 4L | Control + Vehicle vs. CORT +Vehicle  CORT + Vehicle vs. CORT + nor-BNI | Two-way ANOVA | F (1, 20) = 2.939  t (20) = 3.382  t (20) = 3.152 | 0.0176  0.0297 |
| 4M | Control + Vehicle vs. CORT +Vehicle  CORT + Vehicle vs. CORT + nor-BNI | Two-way ANOVA | F (1, 20) = 5.206  t (20) = 3.326  t (20) = 3.549 | 0.0034  0.0020 |
| 4N | Control + Vehicle vs. CORT +Vehicle  CORT + Vehicle vs. CORT + nor-BNI | Two-way ANOVA | F (1, 20) = 1.242  t (20) = 3.104  t (20) = 1.031 | 0.0331  0.8967 |
| 4O | Control + Vehicle vs. CORT +Vehicle  CORT + Vehicle vs. CORT + nor-BNI | Two-way ANOVA | F (1, 20) = 4.170  t (20) = 3.798  t (20) = 2.981 | 0.0011  0.0074 |
| 4P | Control + Vehicle vs. CORT +Vehicle  CORT + Vehicle vs. CORT + nor-BNI | Two-way ANOVA | F (1, 16) = 7.107  t (16) = 8.934  t (16) = 5.041 | < 0.0001  0.0007 |
| 4Q | Control + Vehicle vs. CORT +Vehicle  CORT + Vehicle vs. CORT + nor-BNI | Two-way ANOVA | F (1, 16) = 23.37  t (16) = 4.860  t (16) = 6.348 | 0.0011  < 0.0001 |
| 4R | Control + Vehicle vs. CORT +Vehicle  CORT + Vehicle vs. CORT + nor-BNI | Two-way ANOVA | F (1, 16) = 7.004  t (16) = 5.545  t (16) = 4.471 | 0.0003  0.0023 |
| 5C | Control + AAV-EGFP vs. CORT + AAV-EGFP  CORT + AAV-EGFP vs. CORT + AAV-*Pax6* | Two-way ANOVA | F (1, 36) = 2.597  t (36) = 3.324  t (36) = 3.704 | 0.0122  0.0042 |
| 5D | Control + AAV-EGFP vs. CORT + AAV-EGFP  CORT + AAV-EGFP vs. CORT + AAV-*Pax6* | Two-way ANOVA | F (1, 36) = 5.607  t (36) = 4.183  t (36) = 4.285 | 0.0011  0.0008 |
| 5E | Control + AAV-EGFP vs. CORT + AAV-EGFP  CORT + AAV-EGFP vs. CORT + AAV-*Pax6* | Two-way ANOVA | F (1, 36) = 8.271  t (36) = 4.579  t (36) = 3.442 | 0.0003  0.0089 |
| 5G | Control + AAV-EGFP vs. CORT + AAV-EGFP  CORT + AAV-EGFP vs. CORT + AAV-*Pax6* | Two-way ANOVA | F (1, 16) = 11.48  t (16) = 3.069  t (16) = 3.981 | 0.0432  0.0064 |
| 5H | Control + AAV-EGFP vs. CORT + AAV-EGFP  CORT + AAV-EGFP vs. CORT + AAV-*Pax6* | Two-way ANOVA | F (1, 16) = 15.91  t (16) = 3.691  t (16) = 4.325 | 0.0118  0.0031 |
| 5I | Control + AAV-EGFP vs. CORT + AAV-EGFP  CORT + AAV-EGFP vs. CORT + AAV-*Pax6* | Two-way ANOVA | F (1, 16) = 5.404  t (16) = 3.704  t (16) = 4.609 | 0.0115  0.0017 |
| 5J | Control + AAV-EGFP vs. CORT + AAV-EGFP  CORT + AAV-EGFP vs. CORT + AAV-*Pax6* | Two-way ANOVA | F (1, 16) = 8.835  t (16) = 4.054  t (16) = 4.237 | 0.0055  0.0038 |
| 5K | Control + AAV-EGFP vs. CORT + AAV-EGFP  CORT + AAV-EGFP vs. CORT + AAV-*Pax6* | Two-way ANOVA | F (1, 16) = 30.74  t (16) = 18.63  t (16) = 10.08 | < 0.0001  < 0.0001 |
| 5L | Control + AAV-EGFP vs. CORT + AAV-EGFP  CORT + AAV-EGFP vs. CORT + AAV-*Pax6* | Two-way ANOVA | F (1, 16) = 40.87  t (16) = 8.914  t (16) = 8.984 | < 0.0001  < 0.0001 |
| 5M | Control + AAV-EGFP vs. CORT + AAV-EGFP  CORT + AAV-EGFP vs. CORT + AAV-*Pax6* | Two-way ANOVA | F (1, 16) = 47.22  t (16) = 5.488  t (16) = 8.165 | 0.0003  < 0.0001 |
| 6C | AAV-mCherry vs. AAV-sgRNA-*Pax6* | unpaired t-test | t (20) = 3.758 | 0.0012 |
| 6D | AAV-mCherry vs. AAV-sgRNA-*Pax6* | unpaired t-test | t (20) = 3.047 | 0.0064 |
| 6E | AAV-mCherry vs. AAV-sgRNA-*Pax6* | unpaired t-test | t (20) = 3.995 | 0.0007 |
| 6F | AAV-mCherry vs. AAV-sgRNA-*Pax6* | unpaired t-test | t (8) = 4.033 | 0.0038 |
| 6G | AAV-mCherry vs. AAV-sgRNA-*Pax6* | unpaired t-test | t (8) = 3.196 | 0.0127 |
| 6H | AAV-mCherry vs. AAV-sgRNA-*Pax6* | unpaired t-test | t (8) = 2.357 | 0.0462 |
| 6I | AAV-mCherry vs. AAV-sgRNA-*Pax6* | unpaired t-test | t (8) = 2.739 | 0.0255 |
| 6J | AAV-mCherry vs. AAV-sgRNA-*Pax6* | unpaired t-test | t (8) = 3.756 | 0.0056 |
| 6K | AAV-mCherry vs. AAV-sgRNA-*Pax6* | unpaired t-test | t (8) = 4.328 | 0.0025 |
| 6L | AAV-mCherry vs. AAV-sgRNA-*Pax6* | unpaired t-test | t (8) = 7.584 | <0.0001 |
| S1A | Control vs. CORT | unpaired t-test | t (10) = 4.880 | 0.0006 |
| S1B | Control vs. CORT | unpaired t-test | t (10) = 3.823 | 0.0034 |
| S1C | Control vs. CORT | One-way ANOVA | F (2, 15) = 523.5 | 0.9371 |
| S1D | Control vs. CORT | One-way ANOVA | F (2, 17) = 133.6 | 0.9989 |
| S2A | DTA + Vehicle vs. DTA +TAM | unpaired t-test | t (10) = 1.474 | 0.1712 |
| S2B | DTA + Vehicle vs. DTA +TAM | unpaired t-test | t (10) = 1.663 | 0.1272 |
| S4A | Control + Vehicle vs. CORT +Vehicle  CORT + Vehicle vs. CORT + nor-BNI | Two-way ANOVA | F (1, 16) = 5.001  t (16) = 5.863  t (16) = 3.992 | < 0.0001  0.0011 |
| S4B | Control + Vehicle vs. CORT +Vehicle  CORT + Vehicle vs. CORT + nor-BNI | Two-way ANOVA | F (1, 16) = 24.28  t (16) = 3.419  t (16) = 5.934 | 0.0209  0.0001 |
| S4C | Control + Vehicle vs. CORT +Vehicle  CORT + Vehicle vs. CORT + nor-BNI | Two-way ANOVA | F (1, 16) = 11.88  t (16) = 5.372  t (16) = 3.691 | 0.0004  0.0118 |
| S5B | Control + Vehicle vs. CORT +Vehicle  CORT + Vehicle vs. CORT + nor-BNI | Two-way ANOVA | F (1, 16) = 39.36  t (16) = 8.681  t (16) = 8.412 | < 0.0001  < 0.0001 |
| S5C | Control + Vehicle vs. CORT +Vehicle  CORT + Vehicle vs. CORT + nor-BNI | Two-way ANOVA | F (1, 16) = 0.0826  t (16) = 0.8893  t (16) = 0.0928 | 0.3870  0.9272 |
| S5E | Control + Vehicle vs. CORT +Vehicle  CORT + Vehicle vs. CORT + nor-BNI | Two-way ANOVA | F (1, 16) = 12.08  t (16) = 5.819  t (16) = 4.765 | 0.0002  0.0013 |
| S5F | Control + Vehicle vs. CORT +Vehicle  CORT + Vehicle vs. CORT + nor-BNI | Two-way ANOVA | F (1, 16) = 0.1334  t (16) = 1.067  t (16) = 0.1520 | 0.3016  0.8811 |
|  |  |  |  |  |
| S6C | Control + Con vs. CORT + Con  CORT + Con vs. CORT + Nes-Cre | Two-way ANOVA | F (1, 28) = 2.395  t (28) = 2.885  t (28) = 2.335 | 0.0075  0.0269 |
| S6D | Control + Con vs. CORT + Con  CORT + Con vs. CORT + Nes-Cre | Two-way ANOVA | F (1, 28) = 4.271  t (28) = 4.976  t (28) = 3.643 | < 0.0001  0.0011 |
| S6F | Control + Con vs. CORT + Con  CORT + Con vs. CORT + Nes-Cre | Two-way ANOVA | F (1, 20) = 1.974  t (20) = 3.925  t (20) = 3.342 | 0.0008  0.0032 |
| S6G | Control + Con vs. CORT + Con  CORT + Con vs. CORT + Nes-Cre | Two-way ANOVA | F (1, 20) = 2.170  t (20) = 4.411  t (20) = 2.805 | 0.0003  0.0109 |
| S6H | Control + Con vs. Control + Nes-Cre  CORT + Con vs. CORT + Nes-Cre | Two-way ANOVA | F (1, 20) = 68.79  t (20) = 5.959  t (20) = 5.770 | < 0.0001  < 0.0001 |
| S7A | Control + AAV-EGFP vs. CORT + AAV-EGFP  CORT + AAV-EGFP vs. CORT + AAV-*Pax6* | Two-way ANOVA | F (1, 16) = 5.244  t (16) = 8.211  t (16) = 4.056 | < 0.0001  0.0055 |
| S7B | Control + AAV-EGFP vs. CORT + AAV-EGFP  CORT + AAV-EGFP vs. CORT + AAV-*Pax6* | Two-way ANOVA | F (1, 16) = 32.79  t (16) = 8.469  t (16) = 8.471 | < 0.0001  < 0.0001 |
| S7C | Control + AAV-EGFP vs. CORT + AAV-EGFP  CORT + AAV-EGFP vs. CORT + AAV-*Pax6* | Two-way ANOVA | F (1, 16) = 38.67  t (16) = 4.784  t (16) = 6.536 | 0.0012  < 0.0001 |
| S8A | AAV-mCherry vs. AAV-sgRNA-Pax6 | unpaired t-test | t (8) = 3.500 | 0.0081 |
| S8B | AAV-mCherry vs. AAV-sgRNA-Pax6 | unpaired t-test | t (8) = 4.516 | 0.002 |
| S8C | AAV-mCherry vs. AAV-sgRNA-Pax6 | unpaired t-test | t (8) = 5.035 | 0.001 |
