# Supplementary material for: Kappa-opioid receptor activation promotes depression-like behaviors by suppressing Pax6-dependent adult hippocampal neurogenesis
Source: Transl Psychiatry. 2026 Jul 3;16:496. doi: 10.1038/s41398-026-04230-z (PMC13620123; doi:10.1038/s41398-026-04230-z)
Supplement: Supplementary file 6 [file 41398_2026_4230_MOESM13_ESM.docx]

Figure 2

Figure 3

Figure 4

Figure 5

Figure 6

Figure S6E
